# Advancing the genetic engineering toolbox by combining *AsCas12a* knock-in mice with ultra-compact screening

**DOI:** 10.1038/s41467-025-56282-2

**Published:** 2025-01-30

**Authors:** Wei Jin, Yexuan Deng, John E. La Marca, Emily J. Lelliott, Sarah T. Diepstraten, Christina König, Lin Tai, Valentina Snetkova, Kristel M. Dorighi, Luke Hoberecht, Millicent G. Hedditch, Lauren Whelan, Geraldine Healey, Dan Fayle, Kieran Lau, Margaret A. Potts, Moore Z. Chen, Angus P. R. Johnston, Yang Liao, Wei Shi, Andrew J. Kueh, Benjamin Haley, Jean-Philippe Fortin, Marco J. Herold

**Affiliations:** 1 Olivia Newton-John Cancer Research Institute, Heidelberg, Melbourne, Australia; 2 The Walter and Eliza Hall Institute of Medical Research, Parkville, Melbourne, Australia; 3 Department of Medical Biology, University of Melbourne, Parkville, Melbourne, Australia; 4 The State Key Laboratory of Pharmaceutical Biotechnology, School of Life Sciences, Nanjing University, Nanjing, China; 5 School of Cancer Medicine, La Trobe University, Bundoora, Melbourne, Australia; 6 Department of Molecular Biology, Genentech, Inc., South San Francisco, California, USA; 7 Computational Sciences, Genentech, Inc., South San Francisco, California, USA; 8 Drug Delivery, Disposition and Dynamics, Monash Institute of Pharmaceutical Sciences, Monash University, Parkville, Melbourne, Australia; 9 Present Address: Université de Montréal, Centre de recherche de l’Hôpital Maisonneuve-Rosemont, Rosemont, Canada

**Keywords:** CRISPR-Cas9 genome editing, Genetic engineering, Cancer models

## Abstract

Cas12a is a next-generation gene editing tool that enables multiplexed gene targeting. Here, we present a mouse model that constitutively expresses enhanced *Acidaminococcus sp. Cas12a* (*enAsCas12a*) linked to an mCherry fluorescent reporter. We demonstrate efficient single and multiplexed gene editing in vitro, using primary and transformed cells from *enAsCas12a* mice. We further demonstrate successful in vivo gene editing, using normal and cancer-prone *enAsCas12a* stem cells to reconstitute the haematopoietic system of wild-type mice. We also present compact, genome-wide Cas12a knockout libraries, with four crRNAs per gene encoded across one (Scherzo) or two (Menuetto) vectors, and demonstrate the utility of these libraries across methodologies: in vitro enrichment and drop-out screening in lymphoma cells and immortalised fibroblasts, respectively, and in vivo screens to identify lymphoma-driving events. Finally, we demonstrate CRISPR multiplexing via simultaneous gene knockout (via Cas12a) and activation (via dCas9-SAM) using primary T cells and fibroblasts. Our *enAsCas12a* mouse and accompanying crRNA libraries enhance genome engineering capabilities and complement current CRISPR technologies.

## Abstract (web summary)

Cas12a represents the next generation of gene editing. Here, the authors present the generation and validation of a Cas12a transgenic mouse model. Additionally, the authors create whole-genome Cas12a knockout libraries, and demonstrate their utility across multiple in vitro and in vivo screens.

## Introduction

CRISPR-Cas9, the first developed CRISPR-Cas-based gene editing variant, has found near unparalleled utility in biological and medical research. A particular strength of using CRISPR-Cas9 is the ability to undertake rapid, targeted genetic screens to identify, for example, genes involved in tumourigenesis or clinical drug resistance^1,2^. Cas12a (Cpf1) is an RNA-guided endonuclease that distinguishes itself from Cas9 by its short CRISPR RNAs (crRNAs), intrinsic RNase activity, and different protospacer adjacent motif (PAM) requirements^3^. RNase activity is necessary for Cas12a to extract mature crRNAs from precursor transcripts (pre-crRNA arrays) by recognition of direct repeat (DR) hairpins upstream (5’) of the crRNA targeting sequence. Concatenating multiple guide RNAs within a single pre-crRNA array is therefore possible, and enables combinatorial targeting of one or multiple genes from a compact, easily-clonable, RNA Pol-III expression cassette^4,5^. The gene editing effectiveness of Cas12a has been improved by the engineering of enhanced *Acidaminococcus sp*.-derived *Cas12a* (*enAsCas12a*)^6^, but this was previously only applicable in mammalian cells in vitro. To extend the applications of Cas12a-mediated gene editing, we have generated an *enAsCas12a* knock-in mouse and demonstrated its efficacy when targeting genes, individually or simultaneously, in vitro in primary cells and cell lines, and in vivo via haematopoietic reconstitution. We have furthermore developed two murine-specific, ultra-compact pre-crRNA libraries, enabling highly effective genome-scale CRISPR-Cas12a knockout screens in cells derived from this model in vitro as well as in vivo.

## Results

### Generation and characterisation of the *enAsCas12a* mouse

**Fig. 1:**
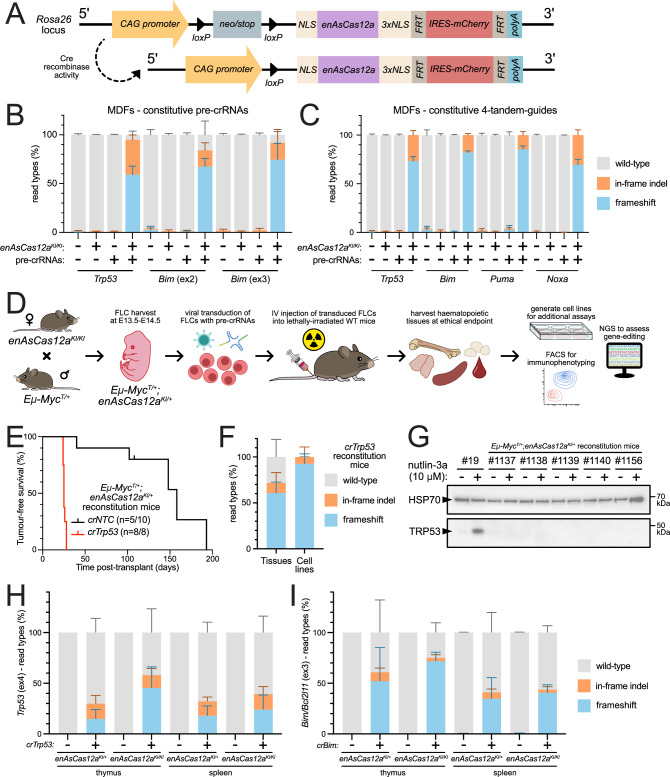
Generation and validation in vitro and in vivo of the *enAsCas12a* knock-in transgenic mouse. **A** Diagram of the *Rosa26*-targeting construct for the genomic insertion of *enAsCas12a*, and the removal of the *neo/stop* cassette to enable constitutive expression. **B** NGS results showing the efficacy of constitutively expressed pre-crRNAs in MDFs (*n* = 3 each (WT and *enAsCas12a*^*KI/KI*^ MDF lines)). Cells were sequenced to assess gene editing at the target gene of the transduced pre-crRNAs. **C** NGS results showing the efficacy of the 4-tandem-guide construct (*n* = 3 each (WT and *enAsCas12a*^*KI/KI*^ MDF lines)). Cells were sequenced to assess gene editing at each of the target genes of the transduced 4-tandem-guide pre-crRNA array. **D** Schematic of the general process for haematopoietic reconstitution of WT mice with *Eμ-Myc*^*T/+*^*;enAsCas12a*^*KI/+*^ cells. **E** Combined survival curve of mice from two independent experiments that underwent haematopoietic reconstitution after transplantation with *Eμ-Myc*^*T/+*^*;enAsCas12a*^*KI/+*^ FLCs, transduced with either *crTrp53* (*n* = 8) or *crNTC* (*n* = 10, with 5 mice having developed lymphoma). **F** NGS results for *Trp53* editing from the tumourous tissue and concomitant lymphoma cell lines from mice (*n* = 7) reconstituted with the *Eμ-Myc*^*T/+*^*;enAsCas12a*^*KI/+*^*;crTrp53* FLCs, demonstrating knockout efficacy. **G** Western blot validating TRP53 loss in independent *Eμ-Myc*^*T/+*^*;enAsCas12a*^*KI/+*^;*crTrp53* cell lines (*n* = 5) derived from the splenic tissue of the reconstituted mice. Positive control cell line #19 was derived from a double-transgenic *Eμ-Myc*^*T/+*^*;enAsCas12a*^*KI/+*^ lymphoma-burdened mouse with a spontaneous *Trp53* mutation. TRP53 stabilisation was induced via 24 h treatment with nutlin-3a (in the presence of QVD-O-Ph). HSP70 expression was used as a loading control. **H**, **I** NGS results showing the efficacy of in vivo gene editing of *Trp53* (**H**) and *Bim/Bcl2l11* (**I**) in the thymus and spleen of animals reconstituted with heterozygous *enAsCas12a*^*KI/+*^ and homozygous *enAsCas12a*^*KI/KI*^ haematopoietic cells transduced with *crNTC*, *crTrp53*, or *crBim* (ex3) (*n* = 4 each). In all graphs, the mean is plotted, and error bars represent SD. Statistical analyses of NGS data can be found in Supplementary Data 4. Source data are provided as a Source Data file. Abbreviations: CAG cytomegalovirus enhancer, promoter of the chicken beta-actin gene, and splice acceptor of the rabbit beta-globin gene, NLS nuclear localisation signal, IRES = internal ribosome entry site, crRNA CRISPR RNA, MDF murine dermal fibroblast, FLCs foetal liver cells, WT wild-type, NTC non-targeting control.

To introduce Cas12a in vivo, we obtained the E174R/S542R/K548R-substitution variant (enhanced) of *Acidaminococcus sp. Cas12a* (*enAsCas12a*; derived from an unclassified *Acidaminococcus* strain (BV3L6))^6^, which has previously been utilised in functional genomic screening^7,8^. The *enAsCas12a* open reading frame was further modified to contain additional nuclear localisation sequences, which can improve enzyme functionality^9^. The *enAsCas12a* cDNA was then cloned into the Cre-recombinase-inducible expression cassette of a previously described mouse *Rosa26*-targeting construct^10^, further modified to include an *IRES-mCherry* marker (instead of *IRES-GFP*). *enAsCas12a* knock-in (*enAsCas12a*^*KI*^) mice were then generated by pronuclear microinjection of this construct into *C57BL/6* one-cell stage embryos. We confirmed *enAsCas12a* insertion by long-range PCR (Supplementary Fig. 1A). Once generated, homozygous *enAsCas12a*^*KI/KI*^ mice were crossed with *CMV-Cre* mice to remove the *loxP*-flanked *neo/stop* cassette (Fig. 1A) and allow constitutive, whole-body *enAsCas12a* expression, as evidenced by measurement of peripheral blood mCherry expression (Supplementary Fig. 1B). This was further demonstrated by mCherry expression in haematopoietic cells/tissues via flow cytometry; almost 100% of peripheral blood cells were mCherry + , and ~80% of haematopoietic organ cells (thymus, bone marrow, spleen, and lymph nodes) had detectable marker fluorescence (Supplementary Fig. 1C). Aware that these mice may be used with *enAsCas12a* in a heterozygous state, we examined whether there was any difference in transgene expression (via mCherry reporter signal) between the haematopoietic organs derived from *enAsCas12a*^*KI/+*^ and *enAsCas12a*^*KI/KI*^ mice. Indeed, we observed ~2-3× greater mCherry fluorescence in the homozygous *enAsCas12a*^*KI/KI*^ tissues compared to the heterozygous *enAsCas12a*^*KI/+*^ tissues (Supplementary Figs. 1D, E). As expected, this difference in *enAsCas12a* expression was also present at the transcriptional level when assessed by qRT-PCR. While *enAsCas12a* expression in homozygous *enAsCas12a*^*KI/KI*^ tissues was largely similar (Supplementary Fig. 1F), comparisons between wild-type (WT), *enAsCas12a*^*KI/+*^, and *enAsCas12a*^*KI/KI*^ tissues clearly showed increased expression *enAsCas12a* in *enAsCas12a*^*KI/KI*^ tissues (Supplementary Fig. 1G).

Lastly, to assess enAsCas12a expression-related toxicity, we analysed haematopoietic compartment cell populations, specifically B, T, and myeloid cell types. No changes were observed compared to WT controls, suggesting constitutive enAsCas12a expression was well-tolerated (Supplementary Figs. 2A–D). Furthermore, no health issues were observed in transgenic mice aged up to 250 days. Together, these data suggest our *enAsCas12a* mouse model is healthy and shows consistent whole-body expression of the *enAsCas12a* transgene, with higher and more broadly detectable signal in homozygous animals.

### Validation of the *enAsCas12a* gene editing efficacy in vitro

As an initial evaluation of enAsCas12a activity, we isolated murine dermal fibroblasts (MDFs) from *enAsCas12a*^*KI/KI*^ mice, and immortalised them via transduction with SV40 large T antigen to enable long term culture. Additional transductions were then performed with individual pre-crRNAs targeting *Trp53* or *Bim/Bcl2l11* (ex3) (written as *crTrp53* and *crBim*, respectively, and hereafter in this style for these and other genes), as well as a multiplexed combination of these pre-crRNAs separated by unique 5’ DRs and contained in a single expression vector (*crTrp53/crBim*) (Table S1). These genes (and many of the others used below) were chosen as they are well understood members of the intrinsic apoptotic pathway, an area of active research in our labs, and we have extensive experience editing these targets via CRISPR-Cas9. Three days post-transduction, next generation sequencing (NGS) revealed strong editing of *Trp53* and *Bim* individually, and similarly strong editing efficiency of both genes when targeted simultaneously (Supplementary Fig. 3A). Simultaneous targeting of *Cd9* and *Cd81* (two non-essential genes in murine cells) using a *crCd9/crCd81* array (Table S1) also returned strong editing efficiency (Supplementary Fig. 3A). We next wanted to assess editing efficiency in non-immortalised primary MDFs, and so isolated them from *enAsCas12a*^*KI/KI*^ or WT mice and transduced them with *crTrp53* or *crBim* (ex2 or ex3) (Table S1). Target disruption efficiency in transduced *enAsCas12a*^*KI/KI*^ primary MDFs was almost 100% for each locus (Fig. 1B). TRP53 loss was also confirmed via western blotting, with no protein observed even 6 h after treatment with nutlin-3a (MDM2 inhibitor, which indirectly leads to TRP53 stabilisation/activation^11^) (Supplementary Fig. 3B).

We then extended our investigations of multiplexed gene editing using enAsCas12a. A 4-tandem-guide construct was designed for simultaneous targeting of *Trp53*, *Bim/Bcl2l11*, *Puma/Bbc3*, and *Noxa/Pmaip1* from a single pre-crRNA expression cassette, with the guides separated by unique 5’ DRs (Table S1). Following lentiviral delivery of this construct in WT and *enAsCas12a*^*KI/KI*^ primary MDFs, we observed 100% editing efficiency for each targeted gene in the latter (Fig. 1C). To expand our assessment of enAsCas12a activity to other cell types and biological contexts, homozygous *enAsCas12a*^*KI/KI*^ female mice were crossed with *Eμ-Myc*^*T/+*^ males (a well-characterised model for B cell lymphoma^12,13^), followed by generation of *Eμ-Myc*^*T/+*^*;enAsCas12a*^*KI/+*^ lymphoma cell lines from the progeny that developed MYC-driven lymphoma. Using lentiviral delivery, we transduced three independent *Eμ-Myc*^*T/+*^*;enAsCas12a*^*KI/+*^-derived cell lines with our constitutively expressed pre-crRNAs and obtained ~50% gene editing efficiency for both *Trp53* and *Bim/Bcl2l11* when individually targeted (Supplementary Fig. 3C). Multiplex targeting was more variable, resulting in ~15% gene editing efficiency for *Trp53*, ~10% for *Bim/Bcl2l11*, and less for both *Puma/Bbc3* and *Noxa/Pmaip1* (Supplementary Fig. 3D). These data suggest that the lower enAsCas12a expression we observed in heterozygotes may correlate with reduced editing efficiency, but there may also be cell-specific differences in enAsCas12a expression and/or activity between primary and transformed cell types.

Previous studies have evaluated the specificity of enAsCas12a in human cell lines^6,7^. To validate specificity of our enAsCas12a model, we assessed editing at potential off-target sites for individual crRNAs in both our *enAsCas12a*^*KI/KI*^ MDF and *Eμ-Myc*^*T/+*^*;enAsCas12a*^*KI/+*^ B lymphoma cell lines. For *crTrp53*, editing was observed at a single position outside of the intended target site (Supplementary Fig. 3E). This site, on chromosome 17, lies within the *transformation related protein 53, pseudogene* (*Trp53-ps*) locus, which shares near-complete sequence congruence with the expressed *Trp53* gene^14,15^. For *crBim* (ex2), several off-targets were predicted. Assessment of the top five revealed background editing of < 10% for all sites (Supplementary Fig. 3F). There were no predicted or observed off-targets for *crBim* (ex3). For the 4-tandem-guide gene targets, in both MDFs and lymphoma cells, *Trp53* and *Bim* (ex2) off-target effects were essentially identical to those observed for their individualised crRNAs (Supplementary Figs. 3G, H), which is logical considering they use the same pre-crRNA sequences. The additional pre-crRNAs included in the 4-tandem vector (targeting *Puma* and *Noxa*) were each predicted to have two off-target sites, and negligible editing was observed for each across all tested conditions (Supplementary Figs. 3I, J).

Collectively, these data demonstrate our *enAsCas12a* model is capable of efficient gene editing in vitro using constitutively expressed pre-crRNA vectors, in both individual and multiplexed configurations, and with minimal off-target effects. However, cell-type and zygosity may impact editing efficacy.

### Validation of *enAsCas12a* gene editing efficacy in vivo

Having established the efficiency of our enAsCas12a system in vitro in both primary and transformed cell lines, our next aim was to evaluate in vivo functionality. To do so, we performed haematopoietic reconstitutions; transducing *Eμ-Myc*^*T/+*^*;enAsCas12a*^*KI/+*^ foetal liver cells (FLCs; these are pre-leukaemic cells that will develop into a monoclonal B cell lymphoma after transplantation^16^) with constitutive *crNTC* (non-targeting control pre-crRNA) or *crTrp53* constructs (GFP + ) ex vivo, then transplanting the transduced FLCs into lethally-irradiated WT (*C57BL/6*) recipient mice (Fig. 1D). Previous studies have established *Trp53* as the dominant tumour suppressor in this context^17–21^. Across two independent experiments, all mice transplanted with *crTrp53*-transduced FLCs developed lymphoma by 28 days post-transplantation (consistent with the latency observed for mice reconstituted with *Eμ-Myc*^*T/+*^ FLCs where *Trp53* is knocked out using CRISPR-Cas9^16,20^) (Fig. 1E). FACS analyses of the haematopoietic tissues revealed the tumours from both *crNTC* and *crTrp53* mice were composed primarily of immature pro-/pre-B cells (B220^+^IgM^-^IgD^-^) (Supplementary Figs. 4A, B). In cell lines derived from the spleens of these mice, successful enAsCas12a-mediated *Trp53* knockout was confirmed by NGS and western blotting (Figs. 1F, G).

To examine whether differences in zygosity also impacted in vivo use of *enAsCas12a*, we performed haematopoietic reconstitutions of recipient WT mice with FLCs either heterozygous or homozygous for *enAsCas12a*, which had been transduced with either *crNTC*, *crTrp53*, or *crBim*. Six weeks post-transplantation, thymic and splenic tissues were harvested from these mice and sequenced to assess the editing of the targeted genes. At harvest, reconstitution efficiency was slightly better in the thymus, with higher levels of donor cells (Supplementary Fig. 4C), while the proportions of cells observed to possess the pre-crRNA expression vector (GFP + ) were largely equal between both genotypes and tissues (Supplementary Fig. 4D). The differences in editing efficiency of *Trp53* (Fig. 1H) and *Bim/Bcl2l11* (Fig. 1I) between the heterozygotes/homozygotes were more prominent in the thymic tissue, but there was overall a noticeable improvement in editing efficiency in the *enAsCas12a*^*KI/KI*^ tissues. Collectively, these findings demonstrate that our enAsCas12a system is effective for in vivo experimentation.

### Creation and functional assessment of the Menuetto and Scherzo libraries for whole-genome knockout screening in vitro and in vivo

Two genome-scale pre-crRNA expression libraries compatible with Cas12a have been described for use in human cells: Humagne^7^, a library where there are 4 unique pre-crRNAs per gene across 2 constructs (dual); and Inzolia^8^, a library which has 4 unique pre-crRNAs per gene and per construct (quad). However, as there are no publicly disclosed equivalents for use in murine systems, we developed two compact, genome-wide, murine-specific pre-crRNA libraries: Menuetto (dual) and Scherzo (quad) (Supplementary Fig. 5). The Menuetto library contains ~44,000 constructs with pre-crRNAs targeting 21,743 genes, along with 500 NTCs, while the Scherzo library contains ~23,000 constructs with pre-crRNAs targeting 21,721 genes plus 500 NTCs (Supplementary Data 1).

**Fig. 2:**
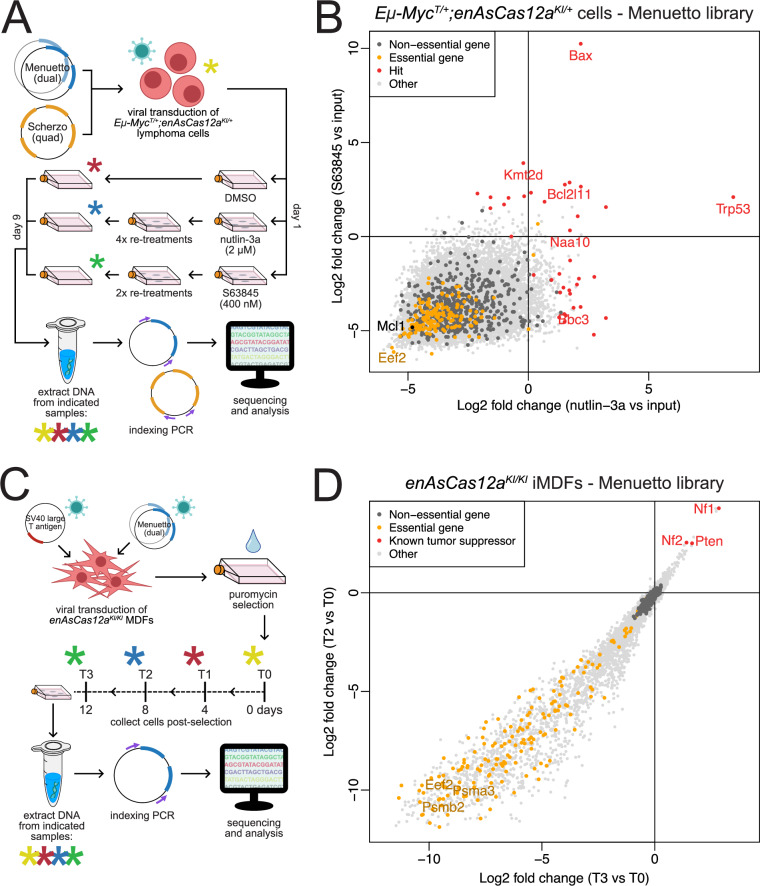
Applications of Cas12a ultra-compact, genome-wide, multiplexed murine-specific pre-crRNA libraries in vitro. **A** Diagram of the design of the whole-genome screens in *Eμ-Myc*^*T/+*^*;enAsCas12a*^*KI/+*^ cells using the Menuetto (dual) and Scherzo (quad) pre-crRNA libraries. *Eμ-Myc*^*T/+*^*;enAsCas12a*^*KI/+*^ lymphoma cells were virally transduced (6 replicates) with each pre-crRNA library. After recovering, cells were treated (day 1) with either DMSO, nutlin-3a (2 μM), or S63845 (400 nM). On day 9, after multiple re-treatments (at the same concentrations), the cells were harvested for DNA. Indexing PCR was performed and then samples underwent NGS. **B** 4-way plot comparing the different arms of the screen samples for the Menuetto library. The *y*-axis compares S63845-treated samples with the input samples, and the *x*-axis compares the nutlin-3a-treated samples to the input samples. Significantly enriched hit genes are indicated in red, essential genes are indicated in orange, non-essential genes are indicated in dark grey, and other genes (genes not classified as either essential or non-essential) are indicated in light grey. A 4-way plot for the Scherzo library screen can be found in Supplementary Fig. 6C. Complete *Eμ-Myc* lymphoma-based screen analyses can be found in Supplementary Data 2. **C** Diagram of the design of the whole-genome drop-out screen in *enAsCas12a*^*KI/KI*^ iMDFs using the Menuetto pre-crRNA library. *enAsCas12a*^*KI/KI*^ MDFs were first immortalised (iMDFs) by viral transduction of SV40 large T antigen, and then iMDFs were virally transduced (3 replicates) with the Menuetto pre-crRNA library to achieve ~300x library coverage. Cells then underwent puromycin selection, before plating them for the screen. Cells were harvested on days 0, 4, 8, and 12 for DNA extraction. Indexing PCR and NGS were then performed. **D** 4-way plot comparing different arms of the iMDF drop-out screen using the Menuetto library. The *y*-axis displays the log2 fold change values for different genes at T2 vs T0, while the *x*-axis displays the log2 fold change values for different genes at T3 vs T0. Essential genes (indicated in orange) and are prominently lost over time. Significantly enriched tumour-suppressor genes are marked in red. Non-essential genes are indicated in dark grey, and other genes are indicated in light grey. Complete iMDF-based drop-out screen analyses can be found in Supplementary Data 3. Abbreviations: MDF murine dermal fibroblast, iMDF immortalised murine dermal fibroblast.

To demonstrate the utility of the Menuetto and Scherzo libraries, we performed genetic screens using one of our aforementioned *Eμ-Myc*^*T/+*^*;enAsCas12a*^*KI/+*^ cell lines (#20). Six replicate transductions were performed for each library before cells were treated with either DMSO, nutlin-3a (2 μM, re-treated 4 times; an MDM2 inhibitor which begets indirect TRP53 activation^11^), or S63845 (400 nM, re-treated 2 times; an inhibitor of the pro-survival BCL-2 family protein MCL-1^22^) (Fig. 2A). Drug concentrations close to the IC_50_ values were chosen, as determined via preliminary viability assays (Supplementary Fig. 6A, B). Once the cells recovered from the multiple treatments, DNA was isolated and NGS was performed to identify enriched pre-crRNAs. Analyses of both the Menuetto (Fig. 2B) and Scherzo (Supplementary Fig. 6C) library screens revealed similar results: strong enrichment of *Trp53*-targeting pre-crRNAs when comparing nutlin-3a-treated cells to the input samples, and strong enrichment of *Bax*-targeting pre-crRNAs when comparing S63845-treated cells to the input samples. This is in line with expectations, as we have previously identified and validated *Trp53* and *Bax* as resistance factors to nutlin-3a- and S63845-mediated killing, respectively, after conducting whole-genome genetic screens using CRISPR-Cas9 in *Eμ-Myc* cells^1,2,23^. By contrast, the DMSO-treated samples showed no standout guide enrichment for either library (complete analyses of both screens can be found in Supplementary Data 2). Furthermore, examination of pre-crRNA loss in the DMSO vs input samples showed strong depletion of pre-crRNAs targeting essential genes for both the Menuetto and Scherzo library screens (Supplementary Figs. 6D, E). We note some predictable drop-outs occur even in the DMSO samples, including pre-crRNAs targeting eukaryotic translation elongation factor 2 (*Eef2*), an essential gene in protein synthesis^24^, and *Mcl-1*, the pro-survival BCL-2 family member upon which *Eμ-Myc* lymphoma cells are known to be most reliant^25^. In agreement with these data, gene set enrichment analysis (GSEA) of these screens indicated that the most strongly enriched gene sets in both the nutlin-3a and S63845 screens are those encompassing various facets of the intrinsic apoptotic pathway (Supplementary Figs. 6F–I), while the most highly depleted gene sets in DMSO vs input samples represent a diverse variety of fundamental cellular processes (Supplementary Figs. 6J, K). We note that the relatively low rate of drop-out hit detection is due to the screen design, which is intended to drive the identification of enriched, drug resistance-promoting gene knockouts. However, drop-out detection is certainly possible in the context of a different screen design (see below). We observed overall higher statistical robustness when screening with the Menuetto (dual) library and, since this format conforms with current standards for large-scale screening efforts like the Cancer Dependency Map Project^26^, we selected the Menuetto library for extended validation.

We next examined the performance of our enAsCas12a model and the Menuetto library for dedicated CRISPR drop-out screening. CRISPR drop-out screening is incredibly useful for identifying perturbation events that influence baseline growth characteristics. However, performing drop-out screens at the whole-genome level is both technically challenging and resource intensive, particularly in primary cell contexts. This is due, at least in part, to the large number of cells required to maintain sufficient coverage ( > 200x) of each guide, which is necessary to infer statistically significant changes in guide presence. The compact size of our Cas12a libraries make them ideal for performing such screens. To demonstrate this utility, we performed a whole-genome drop-out screen in immortalised *enAsCas12a*^*KI/KI*^ MDFs (iMDFs). As described above, MDFs were first immortalised by transduction with SV40 large T antigen and then transduced with the Menuetto library at MOI < 0.5 and library coverage of ~300x, before selecting for transduced cells with puromycin. The iMDFs were then cultured under normal growth conditions and DNA collected from aliquots every 4 days to assess pre-crRNA diversity (Fig. 2C). Analyses revealed significant depletion of pre-crRNAs targeting murine homologues of known essential human genes over time (Figs. 2D, Supplementary Fig. S7A, B), such as *Eef2* (as we saw in the lymphoma screens), as well as *Psmb2* (β4) and *Psma3* (α7), members of the 20S proteasome complex^27^. Separately, pre-crRNAs targeting recognised tumour suppressors (e.g. neurofibromin 1 and 2 (*Nf1* and *Nf2*)) were enriched^28^. *Trp53* was not anticipated, nor was it observed, to be a hit in these assays, as this target is neutralised by the SV40 large T antigen^29^. Additionally, we observed a high concordance in effect sizes between each pre-crRNA per gene-specific pair (Pearson correlation *p* = 0.85). GSEAs served to highlight the depletion of pre-crRNAs targeting a host of core cellular processes (Supplementary Figs. 7C–E).

**Fig. 3:**
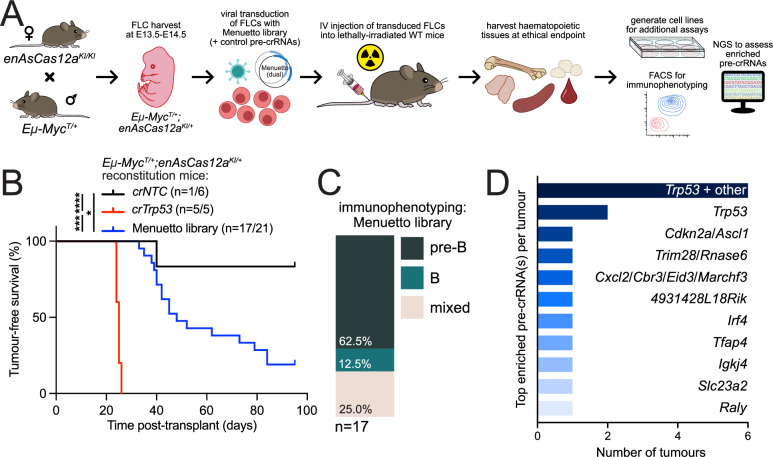
Screening in vivo using the Menuetto library. **A** Diagram of the design of the whole-genome in vivo screen using the Menuetto library and FLCs derived from *Eμ-Myc*^*T/+*^*;enAsCas12a*^*KI/+*^ crosses. **B** Survival curve of mice transplanted with *Eμ-Myc*^*T/+*^*;enAsCas12a*^*KI/+*^ FLCs for in vivo screening with the Menuetto pre-crRNA library. *crNTC* mice remain mostly alive after 95 days, with only one mouse succumbing to pre-B/B cell lymphoma (*n* = 1/6). Two *crNTC* control mice have been excluded from the curve (original cohort *n* = 8) as their death was determined to have a cause other than pre-B/B cell lymphoma (via immunophenotyping). *crTrp53* control mice (n = 5/5) reached ethical endpoint with a median latency of 25 days. Menuetto library mice (n = 17/21) have reached ethical endpoint with a median latency of 48 days. Mantel-Cox log-rank test comparisons: *crNTC* vs Menuetto* p* = 0.0174, *crNTC* vs *crTrp53* *p* = 0.0008, *crTrp53* vs Menuetto *p* < 0.0001. **C** Immunophenotyping results from the tumours of the 17 Menuetto library mice that have so far succumbed to lymphoma. **D** Graph of highly enriched pre-crRNAs in the haematopoietic tissue tumours of the 17 mice obtained so far from the in vivo Menuetto library screen. *Trp53*-targeting pre-crRNAs were the most common, and likely causative even in the instances where multiple, roughly equally represented pre-crRNAs were detected (*Trp53* + other; *n* = 6). Source data are provided as a Source Data file. Abbreviations: crRNA CRISPR RNA, FLCs foetal liver cells, WT wild-type, NTC non-targeting control, NGS next-generation sequencing.

Finally, we sought to examine the effectiveness of the Menuetto library in vivo. Therefore, we again performed haematopoietic reconstitutions using pre-leukaemic *Eμ-Myc*^*T/+*^*;enAsCas12a*^*KI/+*^ FLCs transduced with this library (as well as either *crNTC* or *crTrp53* as controls), and monitored the lethally-irradiated transplanted WT (*C57BL/6*) recipient mice for lymphoma development (Fig. 3A). As expected, *crTrp53* control mice quickly succumbed to lymphoma, while *crNTC* mice developed disease much more slowly (Fig. 3B). Mice transplanted with Menuetto library-transduced *Eμ-Myc*^*T/+*^*;enAsCas12a*^*KI/+*^ FLCs succumbed to lymphoma significantly faster than the *crNTC* mice (Fig. 3B), and immunophenotyping of the tumour cells revealed mostly pre-B cell tumours (Supplementary Fig. 3C). Sequencing of the enriched pre-crRNAs in the tumour tissues revealed *Trp53* was the most common individual hit (*n* = 2/17) (Figs. 3D, Supplementary Fig. 8A). *Trp53* also appeared in combination with other genes (*n* = 6/17), but it is likely that *Trp53* deficiency is the driver of accelerated tumour growth in those circumstances (Figs. 3D, Figure S8A). Demonstrating consistency across methodologies, we re-identified *Tfap4* as a negative regulator of MYC-driven lymphomagenesis, which we had previously observed following a similar Cas9-based screen^20,30^. In addition, we observed enrichment for pre-crRNAs targeting *Irf4*, *Cdkn2a*, and *Slc23a2*, each of which have recognised loss-of-function variants linking them to B cell and other lymphomas^31–35^. Western blotting confirmed TRP53 loss/mutation in tissues from each mouse with an enriched *Trp53* pre-crRNA, and no obvious TRP53 disruptions were observed in mice without *Trp53* pre-crRNA(s) (Supplementary Fig. 8B). Furthermore, we were able to generate and sequence cell lines from most of the lymphoma tissues (*n *= 9/17) and found that, in all instances, pre-crRNA distributions remained largely the same (Supplementary Fig. 8C). While beyond the scope of this current work, we will further characterise the full complement of hits identified from this screen in future studies.

Overall, these screens suggest the Menuetto and Scherzo libraries perform well for compact CRISPR-Cas12a whole-genome screening, capable of being used in a variety of biological contexts – in vitro in both primary and transformed cell lines, and in vivo—and for both positive and negative selection-based screens.

### Combinatorial gene modification: *Cas12a*-mediated gene knockout with *dCas9-SAM*-induced gene expression

**Fig. 4:**
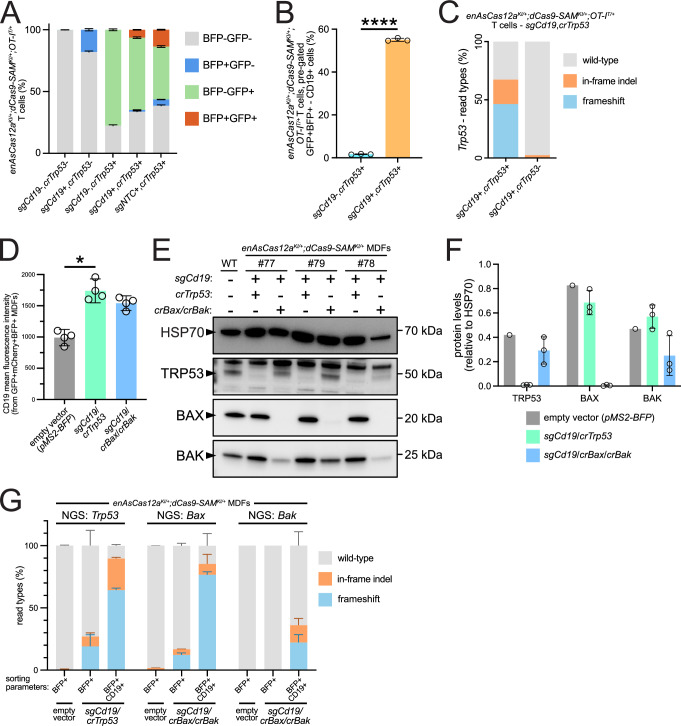
Multiplexing of *enAsCas12a* and *dCas9-SAM* in *OT-I* T cells and MDFs. **A** Quantification of FACS analysis of *enAsCas12a*^*KI/+*^*;dCas9-SAM*^*KI/+*^*;OT-I*^*T/+*^ T cells transduced with an *sgCd19* (BFP-tagged) for dCas9-SAM-mediated gene expression and a *crTrp53* (GFP-tagged) for Cas12a-mediated gene editing (*n* = 3 independent transductions). ~6% of cells from the sample transduced with both constructs were both GFP- and BFP-positive. **B** Quantification of FACS analysis of the T cells transduced with lentiviral vectors expressing *crTrp53* and *sgCd19*, or *crTrp53* and *sgNTC* (*n* = 3 independent transductions each). *sgCd19* was able to induce a statistically significant increase in CD19 expression in the double-transduced cells (Student’s t-test (two-tailed), t = 125.2, df = 4, *p* < 0.0001). **C** NGS data measuring *Trp53* gene editing from the CD19+ populations of T cells transduced with *sgCd19*/*crTrp53*, sorted depending on their GFP status (*n* = 1). **D** Quantification of CD19 mean fluorescence intensity (MFI) in multiplexed *enAsCas12a*^*KI/+*^*;dCas9*^*KI/+*^ MDFs, transduced with a single vector co-expressing *sgCd19* and either *crTrp53* or *crBax/crBak* (*n* = 3 independent transductions). Cells were gated on mCherry, GFP, and BFP expression. Significant upregulation of CD19 was observed in MDFs transduced with the *pMS2-BFP U6_sgCd19 H1_crTrp53* vector (Kruskal-Wallis test with multiple comparisons to the empty vector control, *p* = 0.0225). **E** Western blots demonstrating TRP53, BAX, and BAK loss in the multiplexed *enAsCas12a*^*KI/+*^*;dCas9*^*KI/+*^ MDFs (*n* = 3) or those transduced with the empty vector (*n* = 1). **F** Quantification of the western blots shown in (**E**). Clear loss of both TRP53 and BAX was observed in all samples, but BAK loss was less efficient. **G** NGS results from *enAsCas12a*^*KI/+*^*;dCas9*^*KI/+*^ MDFs (*n* = 3 per transduction), transduced with the *pMS2-BFP* empty vector, or with *pMS2-BFP* containing either *sgCd19/Trp53* or *sgCd19/crBax/crBak*. Cells were sorted based on BFP/CD19 status prior to DNA extraction. Target gene editing was higher in cells that were BFP + CD19+ than in those that were BFP + CD19-. In each graph, the means are plotted, and the error bars represent SD. *p* < 0.05 = *, *p* < 0.0001 = ****. Statistical analyses of NGS data can be found in Supplementary Data 4. Source data are provided as a Source Data file. Abbreviations: MDF murine dermal fibroblast.

Next, we explored the potential for our *enAsCas12a* mouse to be used for inter-Cas multiplexing applications. In addition to facilitating multiplexed gene knockout through pre-crRNA processing, Cas12a can also be multiplexed with orthogonal Cas molecules (such as a dCas9 used for CRISPR activation (CRISPRa)), as its PAM/targeting requirements and guide RNA scaffolds specificities are distinct. To perform such an experiment, we first crossed *enAsCas12a* mice to *OT-I Tg* mice (*OT-I*; in which the T cell receptor is engineered to recognise the immunogen ovalbumin (OVA)^36^), and the resulting progeny were crossed with our previously-described CRISPRa *dCas9-SAM* mice^37^ to generate *enAsCas12a*^*KI/+*^*;dCas9-SAM*^*KI/+*^*;OT-I*^*T/+*^ mice. Splenocytes from these mice were stimulated with OVA peptide and transduced with either a GFP-co-expressing *crTrp53*, a BFP-co-expressing *Cd19*-targeting sgRNA for gene activation (referred to as *sgCd19* hereafter, for this and other genes (Table S2)), or a combination of *crTrp53* and either *sgCd19* or *sgNTC* (also BFP + ) constructs (Supplementary Fig. 9A). Under these conditions, ~6% of cells were successfully transduced with *sgCd19,crTrp53* (~13% for *crTrp53,sgNTC*) (Fig. 4A). For the doubly-transduced samples (GFP + BFP + ), ~55% of *sgCd19,crTrp53* cells expressed CD19, compared to ~1.5% of *sgNTC,crTrp53* cells, demonstrating successful dCas9-SAM-mediated *Cd19* activation (Figs. 4B, Supplementary Fig. 9B). From the CD19+ population of *sgCd19,crTrp53*-transduced cells, we then isolated *crTrp53* + (GFP + ) and *crTrp53*- (GFP-) cells and used NGS to assess the level of *Trp53* gene editing. As expected, we observed no editing of *Trp53* in *sgCd19* + ,*crTrp53*- (GFP-) cells, while *Trp53* was edited with ~50% efficiency in *sgCd19* + ,*crTrp53* + (GFP + ) cells (Figs. 4C, Supplementary Fig. 9C).

The success of this *enAsCas12a*/*dCas9-SAM* multiplexing led us to question whether the technique could be performed using a single transduction. To test this, we cloned *crTrp53*, or an array containing both *crBax* and *crBak*, into the same vector as *sgCd19*. Then, we transduced these vectors into *enAsCas12a*^*KI/+*^*;dCas9*^*KI/+*^ MDFs (or the empty vector as a control) and examined gene activation efficacy by flow cytometry 8 days post-transduction. FACS analysis first demonstrated our live transduced *enAsCas12a*^*KI/+*^*;dCas9*^*KI/+*^ MDFs were triple-fluorescent (mCherry co-expression from *enAsCas12a*, GFP co-expression from *dCas9-SAM*, and BFP co-expression from the crRNA/sgRNA vector) (Supplementary Fig. 10A). In those triple-fluorescent cells, we observed consistent CD19 induction relative to those MDFs transduced with the empty vector (Figs. 4D, Supplementary Fig. 10B). We then examined the efficacy of our gene-knockout via western blotting. For each of the *enAsCas12a*^*KI/+*^*;dCas9*^*KI/+*^ MDFs transduced with the *sgCd19/crTrp53* vector, we observed clear loss of TRP53 expression (Fig. 4E, F). Similarly, for *enAsCas12a*^*KI/+*^*;dCas9*^*KI/+*^ MDFs transduced with the *sgCd19/crBax/crBak* vector, we observed clear loss of BAX, and partial loss of BAK (Fig. 4E, F). To extend the practical potential of this result, we transduced a second set of *enAsCas12a*^*KI/+*^*;dCas9*^*KI/+*^ MDFs (n = 3 per transduction) with the same *sgCd19/crTrp53* or *sgCd19/crBax/crBak* vector (or empty vector), sorted cells based on BFP + CD19- or BFP + CD19+ status, and then performed NGS. While CD19+ cell proportions were not large (Supplementary Fig. 10C), we found that cells isolated based on both BFP + CD19+ status had markedly higher gene editing for *Trp53*, *Bax*, and *Bak*, compared to those isolated based on BFP + CD19- status (Fig. 4G), demonstrating the potential for complex, multi-direction gene editing through the combined use of orthogonal CRISPR mouse model systems.

## Discussion

In this study, we present a murine model featuring constitutive expression of an enhanced *Acidaminococcus*-derived Cas12a (enAsCas12a). We confirmed that sustained enAsCas12a expression is well-tolerated across tissues, particularly in the haematopoietic system, as shown by functional assays and linked expression of an mCherry reporter. Efficacy of enAsCas12a was evaluated using constitutively expressed pre-crRNAs, tested in vitro in both murine dermal fibroblasts (MDFs) and *Eμ-Myc* pre-B/B lymphoma cell lines, and in vivo using mice reconstituted with *Eμ-Myc*^*T/+*^*;enAsCas12a*^*KI/+*^ foetal liver cells. Differences in gene perturbation efficacy correlated with *enAsCas12a* zygosity, though additional cell-type differences (potentially associated with CAG promoter activity) cannot be discounted.

To increase the discovery potential of our *enAsCas12a* mouse, we developed two genome-scale murine-specific Cas12a libraries: the Menuetto library (using dual guide vectors) and Scherzo library (using quad guide vectors), each offering unique benefits over traditional Cas9 sgRNA libraries. The Menuetto library demonstrated robust hit detection and statistical power, likely due to the use of multiple constructs per gene, whereas the Scherzo library’s extremely compact design is ideal for applications reliant on limited cell numbers. Our screens confirmed the efficacy of both libraries: an in vitro survey for factors that suppress lymphoma survival identified expected apoptosis pathway hits (*Trp53*, *Bax*, *Puma*, *Bim*), which have recognised roles in S63845- or nutlin-3a-mediated apoptosis in MYC-driven lymphomas^1,23,38,39^. In addition, this screen uncovered targets with predicted but less-well characterised links to lymphomas: *Naa10* has been reported as necessary for TRP53-mediated apoptosis and was a hit in a similar Cas9-based screen performed by our group^2,40^, while *Kmt2d* has been reported to have tumour suppressive roles in acute myeloid leukaemia^41^ and lung squamous cell carcinoma^42^. We aim to pursue intriguing hits from this screen in future studies. A separate, higher resolution in vitro screen using immortalised *enAsCas12a*^*KI/KI*^ MDFs showed systematic depletion of pre-crRNAs targeting essential genes, along with enrichment of tumour suppressor targets. Lastly, an in vivo screen aimed at uncovering suppressors of MYC-driven lymphomagenesis uncovered genes with established links to cancer formation (e.g. *Trp53*, *Cdkn2a*, *Irf4*), as well as newer targets, like *Tfap4*, which we recently identified in a similar context through Cas9-based screening^20^. As anticipated due to the design of the in vivo screen, we were not able to observe guide depletion for either oncogenes or essential targets. However, the *enAsCas12a* mouse and libraries would be compatible with syngeneic tumour models that could enable studies focused on these classes of targets.

Many phenotypes arise from interactions between multiple genes or pathways, which can only be uncovered by simultaneous, orthogonal gene editing^43,44^. To explore multiplexed genetic engineering applications, we crossed our *enAsCas12a* and previously validated *dCas9-SAM* mice^37^, enabling coincident gene knockout and activation. This configuration uses distinct fluorescent markers – mCherry for Cas12a, GFP for dCas9, and our pre-crRNA/sgRNA expression vectors include a constitutively expressed BFP marker. With this tri-colour approach, FACS can be used to easily enrich for strongly Cas- and guide RNA-positive cells, which will have the highest frequencies of edited alleles. Additionally, the *enAsCas12a* model could be crossed with transgenic mice expressing other CRISPR effectors, including those that enable CRISPR interference^45–47^ or CRISPR base-editing^48^, offering a flexible platform for diverse gene editing applications.

The native ability of Cas12a to process pre-crRNA arrays from a single promoter helps to streamline vector cloning and subsequent multiplexed editing^8,49,50^. To this end, we tested constitutively expressed versions of individual pre-crRNAs and a 4-tandem-guide construct (targeting *Trp53*, *Bim*, *Puma*, and *Noxa* in parallel) in vitro, using primary *enAsCas12a*^*KI/KI*^ MDFs and *Eμ-Myc*^*T/+*^*;enAsCas12a*^*KI/+*^ lymphoma cells. Similar and high levels of gene editing efficacy were observed in the primary MDFs when comparing the individual and 4-tandem-guide configurations, although the lymphoma cells transduced with the 4-tandem-guide construct saw a marked drop in gene editing efficiency, particularly for those pre-crRNAs distal to their promoter. Previous studies have also reported variable efficiencies in either pre-crRNA cleavage or concomitant gene editing when using multiplexed Cas12a pre-crRNAs, and in various species, ranging from bacteria and yeast to human cells^5,51–53^. However, it is notable that we did not observe a reduction in gene editing efficiency using the 4-tandem-guide constructs in homozygous *enAsCas12a*^*KI/KI*^ MDFs, but did see lower *Bak* (positioned 3’ of *crBax* in the *crBax/crBak* array) gene editing in heterozygous *enAsCas12a*^*KI/+*^*;dCas9*^*KI/+*^ MDFs. This is consistent with previous observations for Cas9, where targeting activity correlated with nuclease (Cas) expression^54^, something we have demonstrated can vary based on *enAsCas12a* transgene zygosity.

In summary, our murine *enAsCas12a* system and complementary libraries, Menuetto and Scherzo, provide a powerful platform for genome-wide screens and multiplexed editing of primary systems in vitro and in vivo. By advancing the capabilities of Cas12a-based screens, we aim to enable more comprehensive exploration of genetic interactions and complex biological pathways.

## Methods

### Ethics statement

Care and husbandry of experimental mice was performed according to the guidelines established by both The Walter and Eliza Hall Institute Animal Ethics Committee and the Austin Health Animal Ethics Committee. All ethical endpoint considerations were made by highly trained technicians based on well-established behavioural and physical indicators of lymphoma.

### Animal strains, husbandry, and general procedures

Transgenic *Eμ-Myc*, *enAsCas12a*^*KI*^, *dCas9-SAM*^*KI/+*^, and *enAsCas12a*^*KI/+*^*;dCas9-SAM*^*KI/+*^ mice were maintained on a *C57BL/6-WEHI-Ly5.2* background. *Eμ-Myc* transgenic mice have been described previously^12,13^. *C57BL/6-WEHI-Ly5.1* mice were used as wild-type (WT) control mice and as haematopoietic reconstitution recipients and, alongside the *C57BL/6-OT-I Tg* mice^36^ (RRID:IMSR, JAX:003831), were obtained from The Walter and Eliza Hall Institute breeding facility (Melbourne, Australia). Where necessary, all instances of peripheral blood collection were performed by retro-orbital or cardiac bleeding, and analysed via Advia (Siemens). For all phenotypic assessments, all mice used were between 6-12 weeks old, and age- and sex-matched for each experiment, but sex was otherwise not considered. For all haematopoietic reconstitutions, recipient mice were 7-8 weeks old at time of irradiation and transplantation, and were exclusively male, ensuring that FLCs from both male and female embryos were able to be used.

### *enAsCas12a* transgene creation and transgenic mouse generation

The *pCAG-enAsCas12a(E174R/S542R/K548R)-NLS(nuc)-3xHA (AAS848)* vector^6^ was modified to possess an additional 3× nuclear localisation signals (NLS; *3 × SV40 NLS*) at the C-terminus of the *enAsCas12a* sequence. This modified *enAsCas12a* sequence (and an upstream spacer sequence) was then cloned into the AscI site of a modified *Rosa26*-targeting CTV vector (obtained from Klaus Rajewsky^10^; modified to remove the *pGK-dTA* sequence and replace the *GFP* with *mCherry*). Inducible *enAsCas12a*^*KI*^ mice were generated by pronuclear injection of this *enAsCas12a Rosa26*-targeting vector (7 ng/μL), *Cas9*-targeting sgRNA (10 ng/uL; 5’-CTCCAGTCTTTCTAGAAGAT-3’), and Cas9 protein (50 ng/μL) into *C57BL/6J-WEHI* embryos^55^. Once generated, heterozygous *enAsCas12a*^*KI/+*^ mice were crossed to *CMV-Cre* deleter mice^56^ to remove the *loxP*-flanked *neo/stop* cassette, resulting in the generation of heterozygous mice constitutively expressing *enAsCas12a*, which were bred to produce a homozygous *enAsCas12a*^*KI/KI*^ colony.

### Flow cytometry analyses

To perform FACS on haematopoietic cells/tissues (e.g. peripheral blood samples, bone marrow, thymi, spleens, and lymph nodes), cells were harvested from the mice and processed into single cell suspensions (where necessary). Red blood cells were removed (where necessary – e.g. peripheral blood, spleen) by addition of red cell lysis buffer (made in-house: ammonium chloride (156 mM), sodium bicarbonate (11.9 mM), EDTA (0.097 mM)) before the cells were washed twice with 1 × PBS (Gibco #14190144), centrifuged (526 × *g*), and then resuspended in FACS buffer (1 × PBS, EDTA (5 μM) (Sigma-Aldrich #E8008), 5% FBS (Sigma-Aldrich #12007 C)). To perform FACS on adherent cells (e.g. MDFs), cells were first detached from their plates using cell scrapers (Corning, #3010), placed into FACS buffer, filtered, washed and centrifuged (526 × *g*) using 1 × PBS, before resuspension in FACS buffer. To perform FACS on suspension cells, cells were first filtered, washed and centrifuged (526 × *g*) using 1 × PBS, before resuspension in FACS buffer. As needed, cells were counted using a TC20 Automated Cell Counter (BioRad). Once prepared, single-cell suspensions resuspended in a cocktail of FACS buffer with anti-FCR (made in house; 1:10) and the fluorochrome-conjugated antibodies against proteins of interest. Antibodies used in this study are B220 (RA3-6B2-BV605; 1:200; BioLegend #103244), TCRβ (H57-597-PE-Cy7; 1:400; BioLegend #109222), Mac1 (M1/70-APC-Cy7; 1:400; BD Biosciences #557657), Gr1 (RB68C5-Alexa Fluor 700; 1:400; made in house), IgM (5-1-FITC or 5-1-PE; 1:400; made in house), IgD (11-26 c.2a-BV510; 1:400; BD Biosciences #563110), CD4 (GK1.5-PerCP-Cy5.5; 1:800; BioLegend #100434), CD8 (53.6.7-Alexa Fluor 647; 1:400; made in house), CD19 (ID3-A700; 1:400; made in house), Ly5.1 (A20.1-PE; 1:400; made in-house), and Ly5.2 (S450-Alexa Fluor 700; 1:400; made in-house). Cells were incubated in the antibody cocktail, on ice, for at least 25 m, then washed twice by centrifugation (526 × *g*) with 1 × PBS, and resuspended in FACS buffer for analysis. At the manufacturer-recommended step, and where necessary, viability assessment was performed by staining cells with Zombie UV (BioLegend #423107; diluted 1:1000 in FACS buffer) or ViaDye Red (Cytek #R7-60008; diluted 1:500 in FACS buffer) to mark and exclude dead cells. All flow cytometry samples were analysed using an Aurora (Cytek), FACSymphony A3 (BD Biosciences), or LSR II (BD Biosciences).

### Small-scale pre-crRNA and sgRNA design and cloning

The Cas12a individual pre-crRNAs and 4-tandem-guide array targeting *Trp53*, *Bim/Bcl2l11*, *Bbc3/Puma*, and *Pmaip1/Noxa* were designed using Benchling (sequences given in Table S1). Additional pre-crRNA sequences were pulled from the Menuetto library (full library information given in File S1). All DNA sequences were ordered from either Integrated DNA Technologies (IDT) or Sigma-Aldrich. Individual pre-crRNAs were synthesised with the same DR (5’-TAATTTCTACTCTTGTAGAT-3’) at the 5’ end, which is the sequence recognised by Cas12a, as well as with 4 bp overhangs at the 5’ end for the complementary (5’-TCCC-3’) sequence and reverse complementary (5’-AAAC-3’) sequences. Individual and arrayed pre-crRNAs were cloned into the constitutive lentiviral vector FUGW (Addgene #14883), which had been modified to include a BsmBI restriction site for guide insertion.

For experiments with *enAsCas12a*^*KI/+*^*;dCas9-SAM*^*KI/+*^*;OT-I*^*T/+*^ T cells, *sgNTC* and *sgCd19* were cloned into the BsmBI restriction sites of the WEHI-9.2 vector, after excision of the stuffer sequence (Supplementary Fig. 9D).

The *dCas9-SAM*-compatible *Cd19*-targeting sgRNA vector was generated by first modifying the lenti sgRNA(MS2)_puro optimised backbone (pMS2; Addgene #73797) to remove the puromycin selection sequence and instead incorporate a BFP marker. The *sgCd19* sequence (Table S2) was then cloned into the BsmBI site of the modified *pMS2-BFP* vector, under control of the U6 promoter. The sequences encoding the H1 promoter and *crTrp53* or *crBax/crBak* array were cloned into the BamHI site of the modified *pMS2-BFP U6_sgCd19* vector.

The *sgMyc* sequence was extracted from the LV06 plasmid (Sigma-Aldrich) via PCR (forward primer: 5’-CTATCATATGCTTACCGTAACTTGAAAG-3’, reverse primer: 5’-GTTCGAATTCCATGGGGATCCAAAAAAGCACCGAC-3’) and overnight restriction digest with NdeI and EcoRI. The *pMS2-BFP U6_sgCd19 H1_crTrp53* and *pMS2-BFP U6_sgCd19 H1_crBax/crBak* vectors were also digested with NdeI and EcoRI to remove the *sgCd19* sequence, which was then replaced with the *sgMyc* sequence.

### Cell culture

Primary MDFs were isolated from adult mouse tails. The tail skin (dermis and epidermis) was incubated, with agitation, at 4 °C for 24 h in 1.5 mL DMEM (Gibco #11995065) with dispase II (2.1 U/mL; Merck #D4693). The dermis was then removed from the epidermis and digested with collagenase IV (0.0408 mg/mL; Merck #C5138) at 37 °C for 1 h in 1.5 mL DMEM with 10% foetal bovine serum (FBS; Sigma-Aldrich #12007 C). Single cell suspensions were generated by passing digested dermis through a 100 μm sieve/cell strainer (Falcon #3506) into 3 mL of DMEM with 10% FBS before plating into 6-well tissue culture plates (Falcon #352360). MDFs were maintained in culture using DMEM with 10% FBS, passaged using 1 × trypsin (Lonza #BE02-007E) to dislodge them from the plates, and were cultured for no longer than 3 weeks (not including immortalised MDFs discussed below).

*Eμ-Myc*^*T/+*^*;enAsCas12a*^*KI/+*^ cell lines were derived from spleens of double-transgenic *Eμ-Myc*^*T/+*^*;enAsCas12a*^*KI/+*^ mice after they developed lymphoma (e.g. mice #19, #20, and #23 were euthanised/harvested at the ages of 95, 131, and 105 days old, respectively). *Eμ-Myc*^*T/+*^*;enAsCas12a*^*KI/+*^ cell lines were maintained in cell culture using FMA media^37^, and were cultured for no longer than 3 months.

During their transduction process, FLCs were maintained in foetal liver media: α-MEM with GlutaMax (Gibco #32561037), 10% FBS, HEPES (10 mM; Gibco #15630-080), additional GlutaMax (1x; Gibco #35050-061) sodium pyruvate (1 mM; Gibco #11360-070), and β-mercaptoethanol (50 µM; Sigma-Aldrich #M3148), supplemented with mSCF (0.1 µg/mL; Peprotech #250-03), IL-6 (0.01 µg/mL; made in house), TPO (0.05 µg/mL; Peprotech #315-14), and FLT-3 (0.01 µg/mL; made in house).

HEK293T cells (ATCC #CRL216; RRID: CVCL_0063) were used for virus production and were maintained in cell culture using DMEM with 10% FBS.

*enAsCas12a*^*KI/+*^*;dCas9-SAM*^*KI/+*^*;OT-I*^*T/+*^ splenocytes were plated at ~1 × 10^6^ cells/mL, and cultured in T cell media (RPMI-1640 (Gibco #11875093) containing 10% FBS (Bovogen #SFBS), sodium pyruvate (Gibco #11360070), non-essential amino acids (Gibco #11140050), HEPES (Gibco #15630130), Glutamax (Gibco #35050061), penicillin/streptomycin (Gibco #15140122)), and activated via stimulation with recombinant human IL-2 (100 U/mL; NIH) and SIINFEKL (10 ng/mL; Auspep). After transduction (see below), expanded *enAsCas12a*^*KI/+*^;*dCas9-SAM*^*KI/+*^*;OT-I*^*T/+*^ T cells were washed and maintained in T cell media at 0.5-1 × 10^6^ cells/mL, supplemented with 100 U/mL IL-2.

All cell lines were cultured at 37 °C in 10% CO_2_ and routinely determined to be negative for Mycoplasma infection using a MycoALert detection kit (Lonza #LT07-118).

### Virus production and cell transduction

For transduction of MDF (50,000 cells per transduction) and *Eμ-Myc*^*T/+*^*;enAsCas12a*^*KI/+*^ (100,000 cells per transduction) cell lines, plasmid DNA (10 μg) was packaged using pMDL (5 μg), RSV-Rev (2.5 μg), and VSV-G (3 μg), using an established calcium phosphate precipitation method^57^. Lentiviral supernatant, with the addition of polybrene (8 µg/mL), was added onto MDFs and centrifuged at 1200 × *g* for 45 m at 32°C. *Eμ-Myc*^*T/+*^*;enAsCas12a*^*KI/+*^ cells being transduced with Menuetto/Scherzo library DNA were centrifuged at 1100 × *g* for 2 h at 32°C.

For transduction of FLCs during haematopoietic reconstitutions, plasmid DNA (10 μg) was packaged using pMDL (5 μg), RSV-Rev (2.5 μg), and ENV (5 μg). Lentiviral supernatant was first applied to retronectin (32 µg/mL in 1 × PBS)-coated tissue culture plates (Thermo Fisher Scientific #150200) by centrifugation (2862 × *g*), before the FLCs were applied to the plate, as previously described^20^. Additional foetal liver media was provided, and FLCs were incubated in these virus plates overnight.

For *enAsCas12a*^*KI/+*^*;dCas9-SAM*^*KI/+*^*;OT-I*^*T/+*^ T cells, virus production was performed as described above, but with 60 μg DNA used in the transduction ( ~ 100 × 10^6^ splenocytes). Non-treated 6-well plates were coated with retronectin (48 μg/well) overnight at 4^o^C, washed with 1 × PBS and blocked with 2% BSA for 30 m, before the addition of filtered viral supernatant. For multiplexing, each virus was generated independently before supernatants were combined (1:1). Virus was bound to retronectin-coated plates by centrifugation (2862 × *g*, 2 h, 32^o^C), and the supernatant removed. Activated *enAsCas12a*^*KI/+*^*;dCas9-SAM*^*KI/+*^*;OT-I*^*T/+*^ T cells were then plated onto the virus-coated plates at ~8 × 10^6^ cells/well, before being transduced via incubation for 72 h at 37^o^C.

### Cell sorting

After 2–4 days of recovery post-transduction, MDFs and *Eμ-Myc*^*T/+*^;*enAsCas12a*^*KI/+*^ cells were sorted using an Aria III (BD Biosciences) to acquire only cells with *enAsCas12a*-IRES-mCherry and pre-crRNA-GFP expression.

*enAsCas12a*^*KI/+*^*;dCas9-SAM*^*KI/+*^*;OT-I*^*T/+*^ T cells were sorted using an Aria II (BD Biosciences).

Additional general sorting was also undertaken using an Aria III or an Aria Fusion (BD Biosciences).

### Genomic DNA extraction and next generation sequencing (NGS)

Genomic DNA (gDNA) was extracted from cells using a DNeasy Blood & Tissue kit (QIAGEN #69506), or via incubation in DirectPCR Lysis Reagent (Viagen Biotech #102-T) with proteinase K (Sigma-Aldrich #P4850) (lysis buffer to proteinase ratio 250:1, then incubate at 56 °C for ~1–2 h, then inactivate at 80 °C for 20 m). gDNA samples were prepared for NGS similarly to the previous descriptions^16^. In short, primers that flank the target region of the various pre-crRNAs were designed with 5’ overhangs, and then ordered from IDT (full primer sequences in Table S3). To amplify each gene region, an initial PCR was performed using gDNA (100 ng) with 1 × GoTaq Green Master Mix (Promega #M7123), and 0.5 μM of each overhang primer. Cycling conditions were 18 cycles of 95°C for 2 m, 55-60 °C for 30 s, 70 °C for 30 s. To index the samples for sequencing, a second PCR reaction was then performed using the product from the first PCR (1 μL) with 1 × GoTaq Green and 0.5 μM of each primer (indexing sequences in Table S4). The cycling conditions were 24 cycles of 95°C for 2 m, 55-60 °C for 30 s, and 70 °C for 30 s. Indexed PCR products were pooled and purified using 1.0 × Ampure Beads (Beckman Coulter #A63880), and the pooled samples were sequenced using a MiSeq (Illumina). Indels were quantified using the CRISPR indel calculator (http://crisprindelcalc.net).

### Off-target identification and testing

Potential off-targets for our various individual crRNAs were identified using Cas-OFFinder^58^. Overhang primers to assess each potential off-target were ordered from IDT, and are listed in Table S5. Each sample that was assessed via NGS for on-target editing was also assessed for off-target editing. Where available, 5 off-targets were assessed per candidate crRNA. Indexing PCRs, NGS, and bioinformatic analyses were performed as described above.

### Quantitative reverse transcriptase PCR

Tissues from WT (#351, #352, #353), *enAsCas12a*^*KI/+*^ (#10, #11, #12), and *enAsCas12a*^*KI/KI*^ (#180, #181, #182) mice (*n* = 3 per genotype) were dissected, mechanically homogenised, and placed in 500-1000 μL TRIzol (Thermo Fisher Scientific #15596018). RNA was extracted from TRIzol suspensions as per the manufacturer’s instructions. DNA was digested using an ezDNase kit (Invitrogen #11766051) as per the manufacturer’s instructions. cDNA was synthesised using a SuperScript III First Strand Synthesis System (Thermo Fisher Scientific #11904018) as per manufacturer’s instructions. qRT-PCRs were performed using SYBR Green Universal Master Mix (Applied Biosystems #4309155) and run on a QuantStudio 12 K Flex Real-Time PCR System (Applied Biosystems). Primers were ordered from IDT targeting *Gapdh* (forward: 5’-TGGTGAAGGTCGGTGTGAAC-3’, reverse: 5’-CCATGTAGTTGAGGTCAATGAAGG-3’, as published^59^) and *enAsCas12a* (forward: 5’-GAGCTGAACAGCATCGACCT-3’, reverse: 5’-TCCGCTCATACAGGGCATTC-3’). Primers targeting *enAsCas12a* were confirmed to be specific (i.e. did not detect *dCas9*). No-reverse transcriptase controls were included in the qRT-PCRs and confirmed to have no signal with either primer set. Data were analysed using the ΔΔCt method, normalised to the housekeeping control gene (*Gapdh*) for each sample. Due to the number of samples, multiple qRT-PCR runs were required, and samples were then normalised to an identical positive control sample (cDNA from an *Eμ-Myc*^*T/+*^*;enAsCas12a*^*KI/+*^ cell line) run separately on each plate. Comparisons shown in Supplementary Fig. 1G are also normalised (separately for each tissue) to the *enAsCas12a* expression level in one of the homozygous *enAsCas12a*^*KI/KI*^ samples (from mouse #182).

### Haematopoietic reconstitution

Female *enAsCas12a*^*KI/KI*^ mice were crossed with male *Eμ-Myc*^*T/+*^ mice, and *Eμ-Myc*^*T/+*^*;enAsCas12a*^*KI/+*^ foetal livers from E13.5-14.5 embryos were harvested. Single cell suspensions of FLCs were generated and frozen in freezing medium (90% FBS and 10% DMSO (Sigma-Aldrich #D4540)). As described above, across multiple experiments, *Eμ-Myc*^*T/+*^;*enAsCas12a*^*KI/+*^ FLCs were transduced via spin infection with *crNTC* or *crTrp53* (Table S1), or with the Menuetto whole-genome pre-crRNA library. The next day, these cells were washed using 1 × PBS, filtered, then transplanted into lethally-irradiated (two irradiations of 5.5 Gy, ~3 h apart) 7-8-week-old male *C57BL/6-WEHI-Ly5.1* recipient mice. Tumour-free survival time was defined as the time from FLC transplantation until a reconstituted mouse reached ethical endpoint post-lymphoma development. At ethical endpoint, peripheral blood was collected and analysed via Advia (Siemens), and tumour-burdened tissues were harvested. Immunophenotyping via flow cytometry was used to assess the presence of pre-B/B lymphoma for each mouse, using antibodies against B220, TCRβ, IgM, and IgD (as described above). Mice that were determined to not have succumbed to pre-B/B lymphoma were excluded from further analyses.

Male and female *enAsCas12a*^*KI/KI*^ mice were crossed together, or female *enAsCas12a*^*KI/KI*^ mice to male *C57BL/6* mice to obtain both *enAsCas12a*^*KI/KI*^ and *enAsCas12a*^*KI/+*^ foetal livers from E13.5-14.5 embryos. As above, these FLCs were transduced with *crNTC*, *crTrp53*, or *crBim* (Table S1), and transplanted into lethally irradiated 7-8-week-old male *C57BL/6-WEHI-Ly5.1* recipient mice. After 6 weeks, these mice were euthanised, and their spleen and thymus harvested and prepared as single-cell suspensions for flow cytometry. A portion of the cells were stained for Ly5.1 and Ly5.2 to assess reconstitution success (as described above), and the remainder was sorted based on mCherry+GFP+ status prior to gDNA extraction (as described above).

### Western blot analyses

To inhibit cell demolition, lymphoma cell lines were (where indicated) treated with the pan-Caspase inhibitor QVD-O-Ph (25 μM; MedChemExpress #HY-12305) for 15 min, and then, to induce TRP53 activation, treated with either DMSO (vehicle control) or nutlin-3a (10 μM; MedChemExpress #HY-10029) for 24 h. MDF samples (where indicated) were treated with nutlin-3a (10 μM) for 6 h with no QVD-O-Ph, but otherwise prepared the same way. Cell pellets (either from cell lines or from single-cell suspensions of haematopoietic tissues) were collected and resuspended in RIPA lysis buffer (NaCl (300 mM), SDS (0.2%), Triton X-100 (2%), sodium deoxycholate (1%), Tris HCl (100 mM, pH 8.0)) with protease inhibitor (Roche #11836145001), then incubated on ice for 30 m. Protein-containing supernatant was then collected after centrifugation at 13000 × *g* for 10 m at 4 °C. To quantify protein content, a BCA assay (Thermo Fisher Scientific #23225) was performed, except for the multiplexing experiments (Fig. 4E) where equal cell numbers were used. Then, 25 μg of protein (or the entire sample for the MDF multiplexing experiment, spread across 2 gels) was loaded into a NuPAGE 4 ~ 12% Bis-Tris 1.5 mm gel (Invitrogen #NP0335) and gel electrophoresis performed. Protein was transferred onto a nitrocellulose membrane (Invitrogen #IB23002) according to the manufacturer’s instructions, and the membrane blocked with 5% skim milk powder dissolved in PBS-T (1 × PBS with 0.1% Tween-20 (Sigma-Aldrich #P1379)) for ~1 h at room temperature, and then incubated in primary antibody against P53 (1:2000; Novocastra #NCL-p53-CM5p), β-ACTIN (1:2000; Sigma #A2228), BAX (1:2000; Sigma Aldrich #B9054), BAK (1:2000; Sigma Aldrich #5897), or HSP70 (1:10,000; gift from Dr R Anderson, ONJCRI) (dissolved in PBS-T) at 4°C overnight, with agitation. The next day, the membrane was washed ~3 times with PBS-T before incubation with HRP-conjugated anti-rabbit (Southern Biotech #4010-05) or anti-mouse (Southern Biotech #1010-05) secondary antibody. The protein bands were visualised by adding Immobilon Forte Western HRP substrate (Millipore #WBLUF0100) on a ChemiDoc XRS+ (BioRad).

### crRNA design for the Menuetto library (dual pre-crRNA library)

For each mouse protein-coding gene found in Ensembl Release 102, we designed 2 pairs of 23mer spacer sequences for the enAsCas12a nuclease based on the enAsCas12a design rules implemented in the crisprVerse^60^. First, we filtered out spacer sequences with at least one of the following characteristics: contains a poly-T stretch, has GC content below 20% or above 80%, or contains a recognition site for the restriction enzymes EcoRI and KpnI used for lentiviral cloning. Spacer sequences were then selected to (1) minimise the number of putative off-targets located in other coding sequences, (2) optimise on-target activity using the enPAM+GB prediction algorithm described in ref. ^7^, and (3) target the canonical isoform as defined by Ensembl. We required a minimal distance of 25 nucleotides between spacer sequences within a pair to avoid competing for nuclease occupancy, and required at least 50 nucleotides between pairs as well. When possible, spacers for a given gene were chosen across different exons to increase the probability of having a functional knockout. Spacers located in known Pfam domains, as well as in the first 85% of the CDS region, were prioritised. Finally, to avoid the unintended deletion of functional non-coding elements, we constrained each pair of spacers to regions that do not overlap known non-coding elements described in Ensembl Release 102 (miRNAs, tRNAs, lncRNAs, rRNAs, snRNAs, and snoRNAs). We also included 500 pairs of NTCs. The final library, named Menuetto, contains a total of 46,242 pairs of spacer sequences (full Menuetto library information can be found in Supplementary Data 1). Pre-crRNAs for the Menuetto library (and Scherzo library, see below) were synthesised and cloned (Cellecta) into a modified pLKO.1 vector (Addgene #10878) with pre-crRNAs under control of the hU6 promoter and BFP under control of the EF-1α promoter (Supplementary Fig. 5).

### crRNA design for the Scherzo library (quad pre-cRNA library)

The design of the 4 spacers per gene for the quad pre-cRNA library, named Scherzo, follows the design of the Menuetto library. The 4 spacers per gene are also constrained to a region that does not overlap known non-coding elements. As a result, there is a small number of genes for which it is not possible to design one quad array that targets all isoforms of a given gene without overlapping non-coding elements. For such cases, we designed an additional quad array to target the remaining isoforms. The final library contains a total of 22,839 quad arrays, including 500 NTC quad arrays (full Scherzo library information can be found in Supplementary Data 1).

### In vitro genome-wide Menuetto and Scherzo pre-crRNA library screening and DNA sequencing using *Eμ-Myc* B lymphoma cells

Virus production and *Eμ-Myc*^*T/+*^*;enAsCas12a*^*KI/+*^ cell transduction was performed as described above, with 10 μg library DNA used for each of the six independent transductions undertaken (300,000 cells per transduction) for each library. *Eμ-Myc*^*T/+*^*;enAsCas12a*^*KI/+*^ cells used for screening were not sorted/selected. After recovering from transduction, cell replicates were expanded into T75 flasks (Corning #430641), and treated with either DMSO, nutlin-3a (2 μM, re-treated 4 times; an MDM2 inhibitor, which leads to indirect TRP53 activation), or S63845 (400 nM, re-treated 2 times; an MCL-1 inhibitor). Starting drug concentrations were chosen to be around the IC_50_ value, as determined by viability assays performed on the untransduced parental cell line as previously described^2^ (Supplementary Figs. 6A, B). In short, 30,000 cells were incubated in media with the drug of choice for 24 h, before their viability was assessed by staining with Annexin V-A647 (made in-house, 1:2000) and propidium iodide (1 μg/mL, Sigma-Aldrich #P4170) and measuring live cell proportions via flow cytometry. The first drug treatments occurred ~7 days post-transduction. After recovering from the final treatment, pellets of 4 million cells were collected, washed with 1 × PBS, and the DNA extracted using a DNeasy Blood & Tissue kit (QIAGEN #69506).

Pre-crRNAs were then amplified from 250 ng of DNA using GoTaq Green according to the manufacturer’s instructions, and the following PCR protocol: 3 m at 95°C, [15 s at 95°C, 30 s at 60°C, 30 s at 72°C repeated 35 times], and 7 m at 72°C. The primers used for amplification and indexation of the pre-crRNAs are given in Table S6. For both libraries, the region that is amplified and indexed encompasses the entire pre-crRNA array, and is ~250–350 bp in size (depending on the specific pre-crRNA array and library). PCRs were performed in duplicate for each sample, with each library indexed separately using the same primer combinations. Products were pooled for each library separately, then cleaned up using Ampure XP beads (Beckman Coulter #A63881) and sequenced on a NextSeq 2000 (Illumina) according to the manufacturer’s instructions.

### Statistical analyses for the Menuetto and Scherzo screens in *Eμ-Myc* lymphoma cells

For both the Menuetto and Scherzo screens, reads were mapped to the libraries using MAGeCK v0.5.9^61^ to generate the raw count data. Raw count data were then stored in a standard Bioconductor SummarizedExperiment object^62^ and normalised for sequencing depth. We performed a differential abundance analysis for each pair or quad of pre-crRNAs separately using the popular limma-voom approach^63^. Specifically, we fitted a linear model to the log-CPM values for each pre-crRNA array, using voom-derived observation and quality weights. We performed robust empirical Bayes shrinkage to obtain shrunken variance estimates for each pre-crRNA array, and we used moderated F-tests to compute p-values for each of the two-group comparisons of interest.

For the Menuetto screen, we obtained gene-level statistics by aggregating statistics per gene from each gene-specific pre-crRNA pair. In particular, we used the fry gene-set enrichment analysis method implemented in limma, and considered the two pairs targeting a given gene as a gene set. This allows the detection of genes that are consistently enriched or depleted for the two pre-crRNA pairs. We applied the Benjamini-Hochberg procedure to obtain an FDR-corrected p-value for each gene. Essential/non-essential gene lists were obtained from previously published work^64^. Hits were selected by using an FDR threshold of 20%. Depletion of pre-crRNAs targeting essential genes was measured by comparing log-fold change between the DMSO-treated and pre-treatment input samples.

For the Scherzo screen, the quad array p-values were corrected for multiple comparisons using the Benjamini-Hochberg procedure. Hits were selected by using an FDR threshold of 5%.

To perform gene set enrichment analysis (GSEA) for both the Menuetto and Scherzo screens, a hypergeometric test was used on enriched genes (logFC > 0) in the nutlin-3a treatment groups. Gene ontology (GO) data for murine gene sets was obtained from the MSigDB database^65^. In the same manner, GSEA was also performed for depleted genes in the DMSO group using a logFC threshold of -5.

### In vitro genome-wide Menuetto pre-crRNA library drop-out screening and DNA sequencing using immortalised MDFs

Primary MDFs were first generated from *enAsCas12a*^*KI/KI*^ mice as described above, and then immortalised (iMDFs) via lentiviral transduction of SV40 large T antigen, carried out as described above for the transduction of MDFs with plasmid DNA (and as previously described^66^). These iMDFs were then lentivirally transduced with the Menuetto library (also as described above), in triplicate, with a multiplicity of infection (MOI) < 0.5, aiming to obtain no more than one pre-crRNA per cell, and also aiming for at least 300 × pre-crRNA coverage. Cell numbers were sufficient to maintain 300 × pre-crRNA coverage was across the whole screen process. Library transduced iMDFs were selected for using puromycin (2.5 µg/mL) for 2 days, and timepoint 0 (T0 = 0 d post-puromycin recovery) samples were then collected for DNA. Cells were cultured under normal culturing conditions without additional stress for 12 days, passaged every 2 days, and samples containing enough cells to obtain 300 × pre-crRNA coverage were harvested every 4 days (T1 = 4 d, T2 = 8 d, T3 = 12 d post-puromycin recovery). Genomic DNA was isolated from cell samples using a Puregene Cell Kit (QIAGEN #158767). Pre-crRNAs were amplified from 96 µg DNA of each sample using Ultra II Q5 Master Mix (New England Biolabs #M0544) according to the manufacturer’s instructions and the following PCR protocol: 30 s at 98°C, [22 cycles of 98°C for 10 s, 66°C for 75 s], and 5 m at 66°C. Primers used for pre-crRNA amplification and indexation are given in Table S6, with 3 unique library indexes and 8 reactions per sample. Amplified and indexed PCR products were pooled and purified using 1.0 × Ampure Beads (Beckman Coulter #A63880), and sequenced using a NextSeq 2000 (Illumina) and NextSeq 2000 P4 XLEAP-SBS Reagents (100 cycles) according to the manufacturer’s instructions.

### Statistical analyses for the Menuetto screen in immortalised MDFs

The statistical analyses for the immortalised MDFs are identical to the analysis of the Menuetto screen performed in *Eμ-Myc* lymphoma cells described above. For the GSEA of genes conferring a growth advantage in the screen, we used a logFC threshold of 0.5.

### In vivo genome-wide Menuetto pre-crRNA library screening, DNA sequencing, and bioinformatics

FLCs from *Eμ-Myc*^*T/+*^*;enAsCas12a*^*KI/+*^ mice were transduced with *crNTC*, *crTrp53*, or the Menuetto library (as described above), and then WT recipient mice transplanted with these cells (as described above). At ethical endpoint after lymphoma development, tumour-burdened tissues were collected and processed to extract DNA for NGS (as described above). NGS data was analysed by using MAGeCK v0.5^61^ to map pre-crRNA reads to the Menuetto library. Genes targeted by the top 4 most abundant pre-crRNAs (by read count) were then plotted in Prism 10 (GraphPad).

### Statistics and reproducibility

Statistical analyses (excluding the analysis of the Menuetto/Scherzo screens described above) and data graphing were performed using Prism (v10.1.1, GraphPad). Statistical analyses for NGS data, performed via two-way ANOVA with Šídák’s multiple comparisons test, are presented in Supplementary Data 4, alongside analyses of other stacked bar data. All statistical analyses performed reflect comparisons between distinct samples, rather than repeated measures. Statistical tests used in each experiment are indicated in their respective figure legends, having been confirmed to conform with normality assumptions prior. Data are presented as means ± standard deviation (SD), and statistical significance between groups is assessed by P-values, denoted by asterisks (* = *p* < 0.05, ** = *p* < 0.01, *** = *p* < 0.001, **** = *p* < 0.0001, and ns = no significant difference). No statistical method was used to predetermine sample sizes. The only data excluded from analyses were mice that reached ethical endpoint but were determined to not have been euthanized due to lymphoma, which we have indicated in the relevant figure legends. Animal distribution into experimental groups was randomised, after controlling for age or sex matching. Mice were monitored by experienced animal technicians who were blinded to the nature of the experiment.

### Reporting summary

Further information on research design is available in the Nature Portfolio Reporting Summary linked to this article.

## Supplementary information

Supplementary Information

Description of Additional Supplementary Files

Supplementary Data 1

Supplementary Data 2

Supplementary Data 3

Supplementary Data 4

Reporting Summary

Transparent Peer Review file

## Source data

Source Data

## Data Availability

Library and screening data is available in Supplementary Material. Source data are provided as a Source Data file. Raw sequencing data is available on the NCBI GEO repository GSE285778. We believe all necessary data has been made available, but if any additional data is desired by a reader, it will be made available as quickly as possible upon request to the corresponding author, with no conditions made on access. Source data are provided with this paper.
