# Supplementary material for: Advancing the genetic engineering toolbox by combining AsCas12a knock-in mice with ultra-compact screening
Source: Nat Commun. 2025 Jan 30;16:974. doi: 10.1038/s41467-025-56282-2 (PMC11782673; doi:10.1038/s41467-025-56282-2)

Supplementary Figure 1

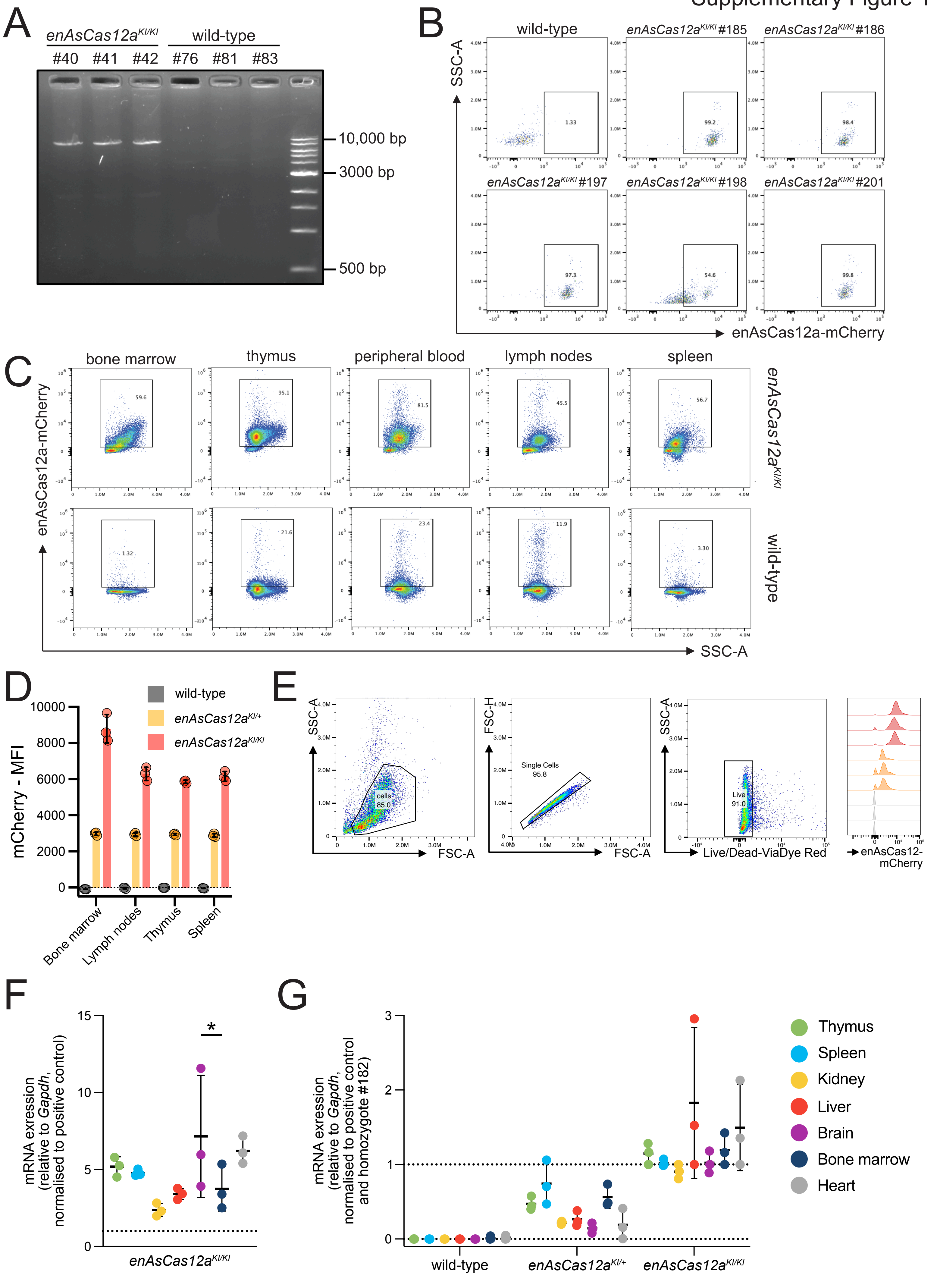

Supplementary Figure 1. Validation of enAsCas12a presence/expression in the transgenic mouse model. (A) Long range PCR of *enAsCas12a* construct presence in *enAsCas12a*$^{KI/KI}$ transgenic mice. The expected band size is 10,126 bp. Samples from *enAsCas12a*$^{KI/KI}$ transgenic mice and WT control mice (*C57BL/6*) are shown (n=3 each). (B) The expression of enAsCas12a-linked mCherry in peripheral blood was detected by FACS. Data from one WT (*C57BL/6*) mouse and 5 enAsCas12a$^{KI/KI}$ transgenic mice are shown. (C) Haematopoietic organ-specific expression of enAsCas12a-linked mCherry was also detected by FACS. Data from representative WT (*C57BL/6*) and *enAsCas12a*$^{KI/KI}$ mice (n=1 each) are shown. (D) Quantification of mCherry mean fluorescence intensity (MFI) of various haematopoietic tissue cells obtained from WT, heterozygous *enAsCas12a*$^{KI/+}$, and homozygous *enAsCas12a*$^{KI/KI}$ animals (n=3 each). (E) Representative FACS plots (from bone marrow) demonstrating the gating strategy used to identify mCherry+ cells and assess MFI. (F) qRT-PCR data comparing the expression level of *enAsCas12a* in homozygous animals (n=3) between different tissues. Expression is measured relative to housekeeping gene *Gapdh*, and was also normalised to a positive control sample. Differences in *enAsCas12a* expression between brain and bone marrow were statistically significant (one-way ANOVA with Kruskal-Wallis multiple comparisons test, $p=0.0333$). All other statistical comparisons were non-significant. (G) qRT-PCR data comparing *enAsCas12a* expression levels between different tissues from WT, *enAsCas12a*$^{KI/+}$, and *enAsCas12a*$^{KI/KI}$ animals (n=3 each). Expression is measured relative to housekeeping gene *Gapdh*, and was also normalised first to a positive control sample, and then (separately for each tissue) to the *enAsCas12a* expression level in one of the homozygous *enAsCas12a*$^{KI/KI}$ samples. Statistical analyses for G can be found in Supplementary File 4. In each graph, the mean is indicated (black bar), and the error bars represent SD. $p<0.05$ = *. Source data are provided as a Source Data file. Abbreviations: MFI = mean fluorescence intensity.

Supplementary Figure 2

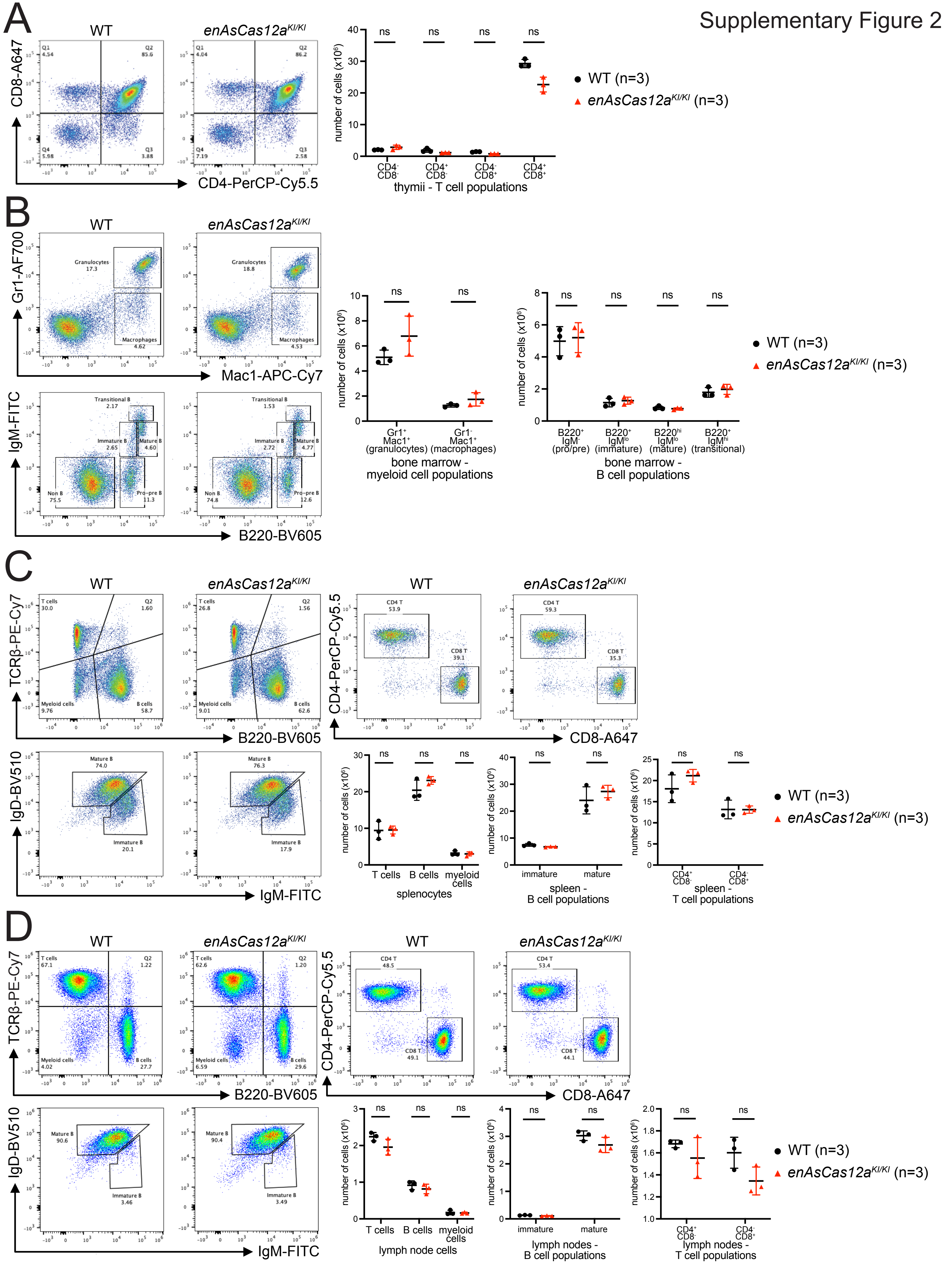

Supplementary Figure 2. *enAsCas12a$^{KI/KI}$* mice exhibit no defects in their haematopoietic system. (A) Representative FACS plots for, and quantification of, T cell distribution between the thymi of *enAsCas12a$^{KI/KI}$* mice (n=3) and WT mice (n=3). (B) Representative FACS plots for, and quantifications of, B cell (pro-B/pre-B = $B220^{+}IgM^{-}$; immature B = $B220^{+}IgM^{lo}$; mature B = $B220^{hi}IgM^{lo}$; transitional B = $B220^{+}IgM^{hi}$) and myeloid cell (macrophages = $Mac1^{+}Gr1^{-}$; granulocytes = $Mac1^{+}Gr1^{+}$) distributions in the bone marrow of *enAsCas12a$^{KI/KI}$* mice (n=3) and WT mice (n=3). (C) Representative FACS plots for, and quantifications of, T cell ($TCR\beta^{+}B220^{-}$; subsets identified by CD4 and CD8) and B cell ($TCR\beta^{+}B220^{+}$; subsets identified by IgM and IgD) distribution in the spleens of *enAsCas12a$^{KI/KI}$* mice (n=3) and WT mice (n=3). (D) Representative FACS plots for, and quantifications of, T cell and B cell distributions specifically from lymph nodes of *enAsCas12a$^{KI/KI}$* mice (n=3) and WT mice (n=3), each identified/characterised as above. For each of these data sets, statistical analyses were undertaken via multiple Mann-Whitney tests with Holm-Šídák multiple comparisons. In each instance, the adjust p-value was >0.05, indicated no significant difference (n.s.) was present between the wild-type and *enAsCas12a$^{KI/KI}$* samples. In each graph, the means are plotted, and the error bars represent SD. Source data are provided as a Source Data file. Abbreviations: WT = wild-type.

Supplementary Figure 3

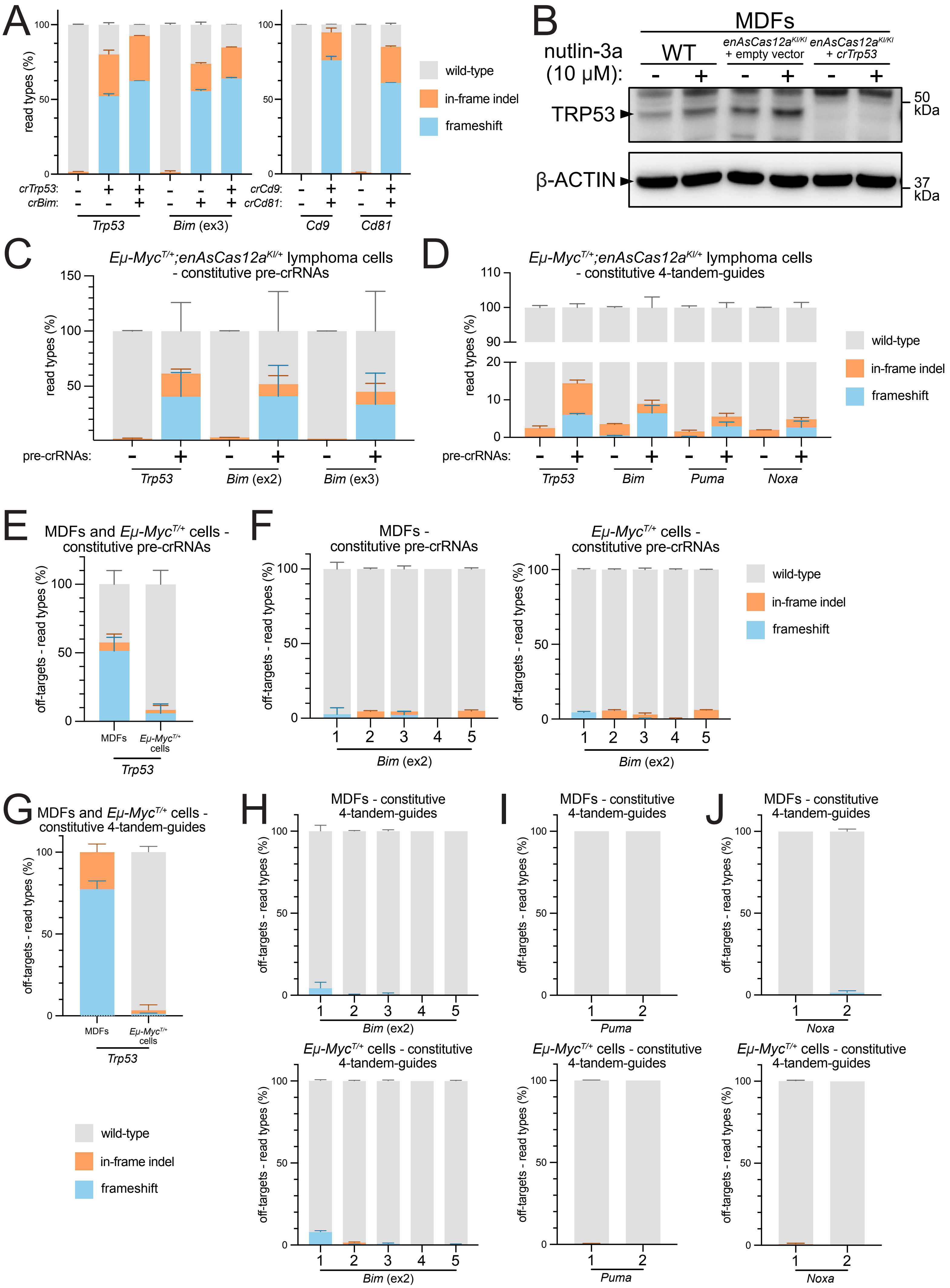

Supplementary Figure 3. Validation of enAsCas12a efficacy in *Eμ-Myc$^{T/+}$;enAsCas12a$^{KI/+}$* cell lines and off-target testing. (A) NGS results showing *Trp53* and *Bim* editing separately and simultaneously (left panel), and simultaneous editing of Cd9 and Cd81 (right panel), in immortalised *enAsCas12a$^{KI/KI}$* MDFs (n=2). (B) Western blotting for TRP53, with β-ACTIN expression used as a loading control. MDFs (n=1 per genotype) were treated for 6 h with nutlin-3a to induce TRP53 stabilisation. No TRP53 expression was observed in *enAsCas12a$^{KI/KI}$* MDFs with *crTrp53* expression, unlike in WT MDFs, or in *enAsCas12a$^{KI/KI}$* MDFs with the empty vector. (C) NGS results showing the efficacy of constitutively expressed pre-crRNAs targeting *Trp53* or *Bim/Bcl2l11* in *Eμ-Myc$^{T/+}$* (n=3) or *Eμ-Myc$^{T/+}$;enAsCas12a$^{KI/+}$* (n=2-3) lymphoma cells. (D) NGS results showing the efficacy of the constitutive 4-tandem-guide construct with pre-crRNAs for parallel targeting of *Trp53*, *Bim/Bcl2l11*, *Puma/Bbc3*, and *Noxa/Pmaip1* in *Eμ-Myc$^{T/+}$* (n=3) or *Eμ-Myc$^{T/+}$;enAsCas12a$^{KI/+}$* (n=3) lymphoma cells. (E) NGS results from off-target amplicons for *Trp53*. While MDFs and *Eμ-Myc$^{T/+}$* lymphoma cells targeted with *crTrp53* (n=3 each) showed ~50% frameshift mutations in the off-target region, bioinformatic analyses revealed this gene-editing was actually occurring in the well-known *Trp53* pseudogene (*Trp53-ps*) [14, 15]. (F) NGS results from 5 off-target amplicons for *crBim* (ex2) in MDFs and *Eμ-Myc$^{T/+}$* lymphoma cells (n=3 each). Minimal off-target indels were observed for this crRNA in MDFs. (G-J) NGS results from off-target amplicons for the genes targeted by the 4-tandem-guide construct in both or *enAsCas12a$^{KI/KI}$* MDFs and *Eμ-Myc$^{T/+}$;enAsCas12a$^{KI/+}$* cells (n=3 each). *Trp53* (G) and *Bim* (H) off-target editing was similar to their individual pre-crRNA constructs. *Puma* (I) and *Noxa* (J) off-target editing was negligible. In each graph, the means are plotted, and the error bars represent SD. Statistical analyses of NGS data can be found in Supplementary File 4. Source data are provided as a Source Data file. Abbreviations: crRNA = CRISPR RNA; WT = wild-type; MDF = murine dermal fibroblast.

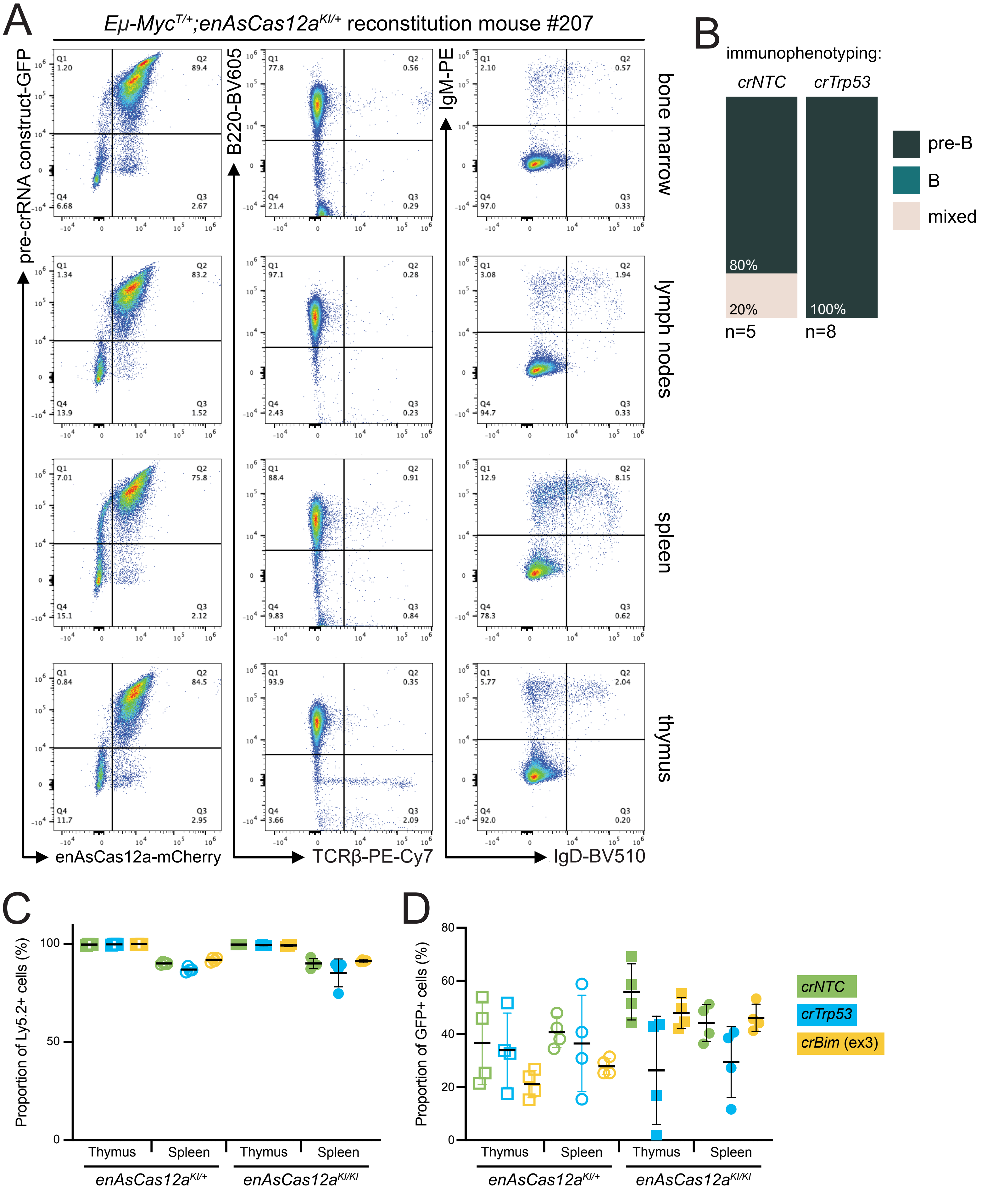
A
Eμ-Myc^T/+;enAsCas12a^KI/+ reconstitution mouse #207
pre-crRNA construct-GFP
enAsCas12a-mCherry
B220-BV605
TCRβ-PE-Cy7
IgM-PE
IgD-BV510
bone marrow
lymph nodes
spleen
thymus
B
immunophenotyping:
crNTC
crTrp53
pre-B
B
mixed
80%
20%
100%
n=5
n=8
C
Proportion of Ly5.2+ cells (%)
Thymus
Spleen
enAsCas12a^KI/+
enAsCas12a^KI/KI
D
Proportion of GFP+ cells (%)
crNTC
crTrp53
crBim (ex3)

Supplementary Figure 4. enAsCas12a efficacy testing *in vivo*. (A) Flow cytometry gating of haematopoietic cells from a representative mouse (experiment performed for n=3 mice total) reconstituted with *Eμ-Myc*$^{T/+}$*;enAsCas12a*$^{KI/+}$*;crTrp53* FLCs. GFP indicates pre-crRNA presence. (B) Immunophenotyping data from haematopoietic organs derived from mice transplanted with *Eμ-Myc*$^{T/+}$*;enAsCas12a*$^{KI/+}$*;crNTC* FLCs (n=5) or *Eμ-Myc*$^{T/+}$*;enAsCas12a*$^{KI/+}$*;crTrp53* FLCs (n=8). Tumours were classified based on B cell antigen status: pre-B ($B220^+IgM^-IgD^-$), B ($B220^+IgM^+IgD^-$ or $B220^+IgM^+IgD^+$), or mixed ($B220^+IgM^-$ and $B220^+IgM^+$). (C) Plot quantifying the percentage of Ly5.2+ cells (donor cells) in the spleens and thymi of heterozygous *enAsCas12a*$^{KI/+}$ and homozygous *enAsCas12a*$^{KI/KI}$ mice (n=4 each). (D) Plot quantifying the percentage mCherry+ (*enAsCas12a*) cells that are also GFP+ (pre-crRNA expression vector) in the spleens and thymi of heterozygous *enAsCas12a*$^{KI/+}$ and homozygous *enAsCas12a*$^{KI/KI}$ mice (n=4 each). In each graph, the means are plotted, and the error bars represent SD. Source data are provided as a Source Data file. Abbreviations: NTC = non-targeting control.

Supplementary Figure 5

A

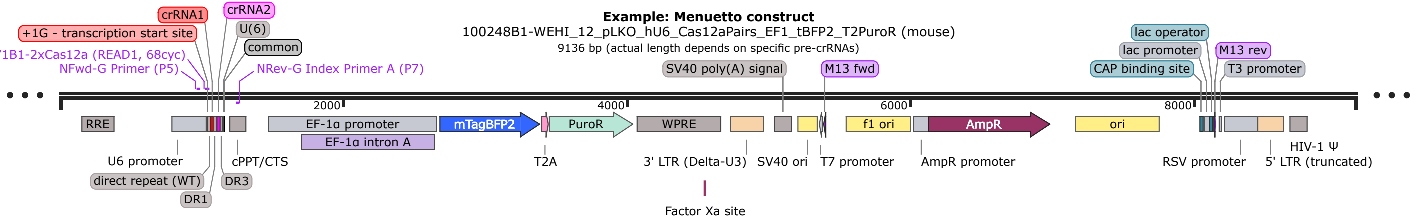

B

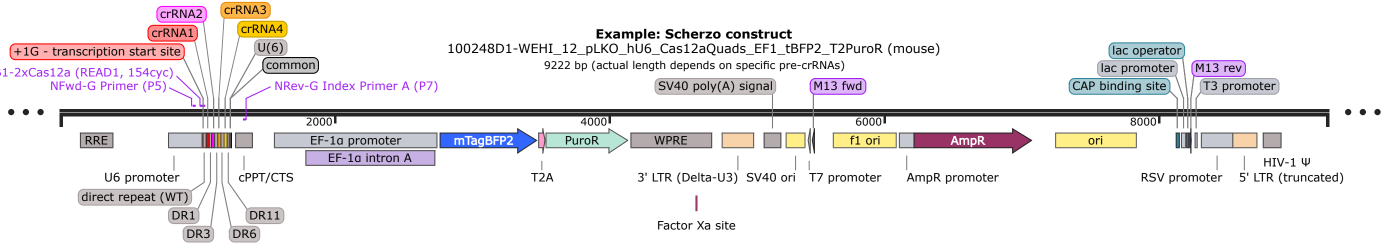

Supplementary Figure 5. Menuetto and Scherzo library constructs. (A, B) Linearised vector maps of example constructs from the Menuetto (A) and Scherzo (B) pre-crRNA libraries. Both constructs are essentially identical, apart from the pre-crRNA + direct repeat cassette. Abbreviations: crRNA = CRISPR RNA; DR = direct repeat.

Supplementary Figure 6

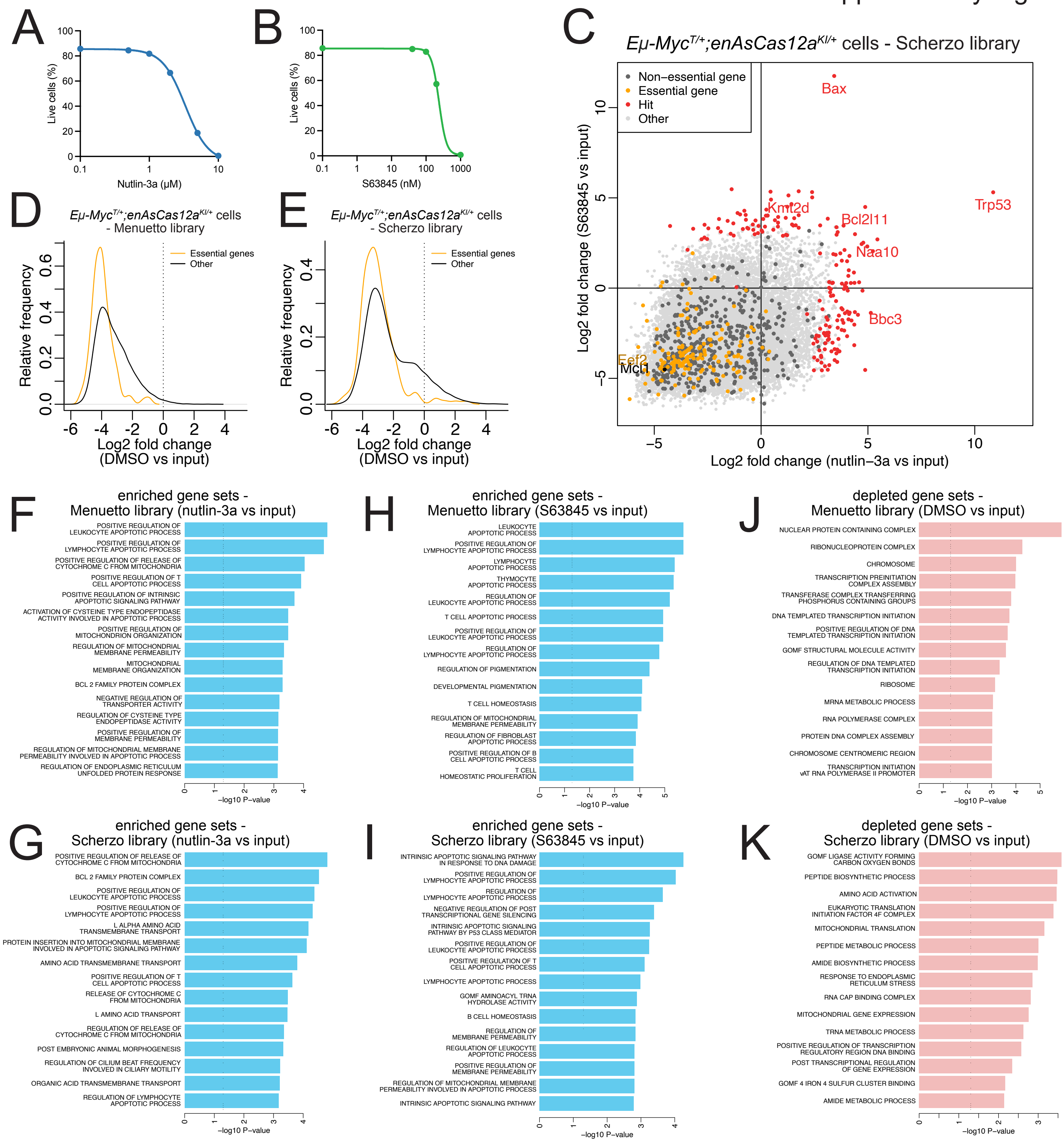

Supplementary Figure 6. Additional *in vitro* lymphoma Menuetto and Scherzo screening data. (A, B) Preliminary viability assays used to determine suitable nutlin-3a (A) and S63845 (B) concentrations used in the Menuetto and Scherzo screens in *Eμ-Myc*$^{T/+}$*;enAsCas12a*$^{KI/+}$ cells. A dose close to the IC_{50} value was selected for each screen. Viability assays were performed once, with technical replicates performed in duplicate. (C) 4-way plot comparing the different arms of the screen samples for the Scherzo library. The *y*-axis compares S63845-treated samples with the input samples, and the *x*-axis compares the nutlin-3a-treated samples to the input samples. Significantly enriched hit genes are indicated in red, essential genes are indicated in orange, non-essential genes are indicated in dark grey, and other genes are indicated in light grey. Complete *Eμ-Myc* lymphoma-based screen analyses can be found in Supplementary File 2. (D, E) Measurement of pre-crRNA drop-out in the Menuetto (D) and Scherzo (E) library screens, comparing the DMSO-treated and input samples, showing the majority of pre-crRNAs, and particularly those targeting essential genes, are lost over the course of the screens. (F, G) GSEA graphs for strongly enriched pre-crRNA target genes from the Menuetto (F) and Scherzo (G) library screens using nutlin-3a. Genes involved in apoptosis are highly ranked. (H, I) GSEA graphs for strongly enriched pre-crRNA target genes from the Menuetto (H) and Scherzo (I) library screens using S63845. Genes involved in apoptosis are highly ranked. (J, K) GSEA graphs for strongly depleted pre-crRNA target genes from the Menuetto (J) and Scherzo (K) library screens using DMSO. Genes regulating fundamental biological processes are most prominent. Source data are provided as a Source Data file. Abbreviations: DMSO = dimethyl sulfoxide

Supplementary Figure 7

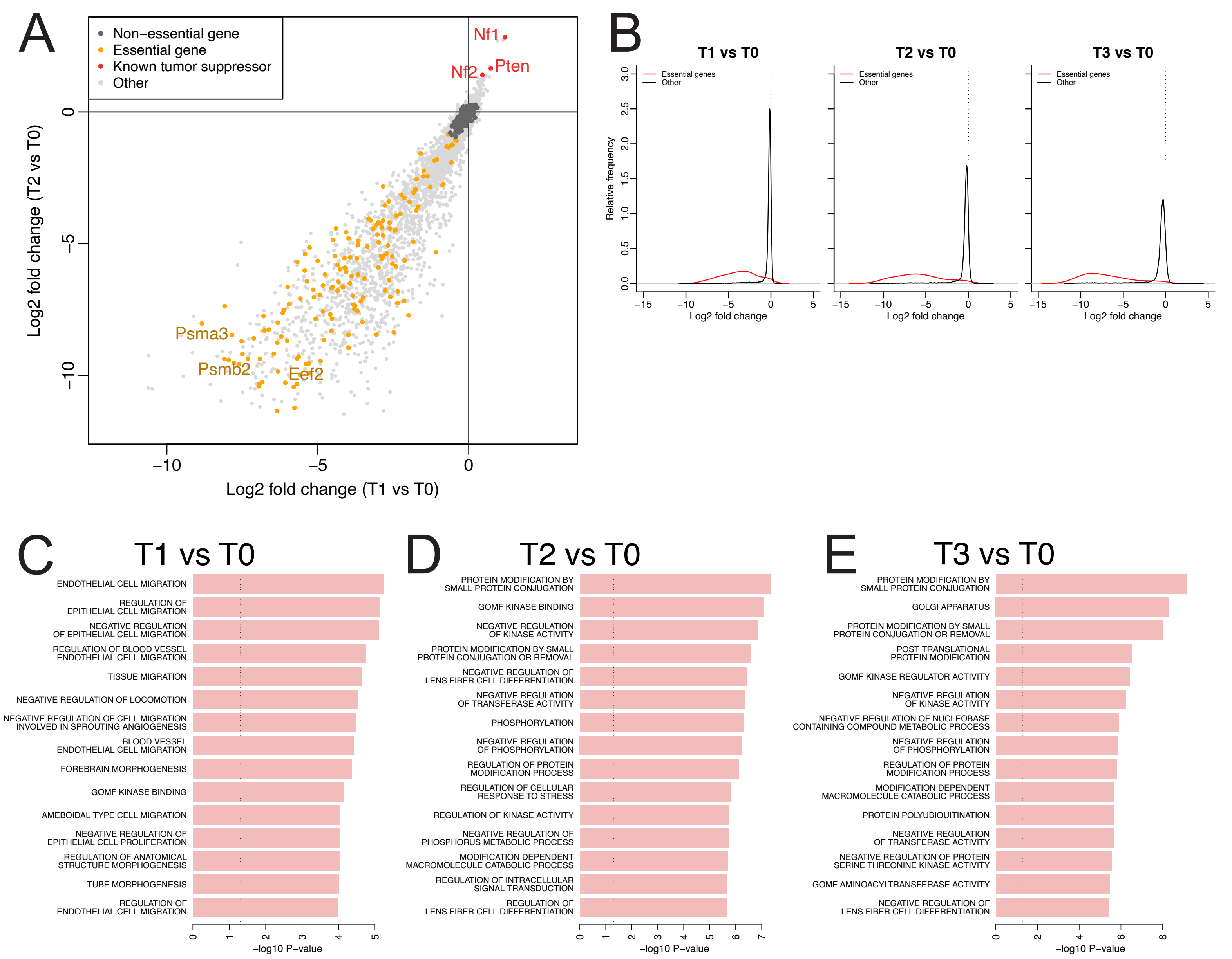

Supplementary Figure 7. Additional *in vitro* MDF Menuetto screening data. (A) 4-way plot comparing different arms of the MDF drop-out screen samples using the Menuetto library. The *y*-axis displays the log2 fold change values for different genes at T2 vs T0, while the *x*-axis displays the log2 fold change values for different genes at T1 vs T0. Essential genes (indicated in orange) and are prominently lost over time. Significantly enriched tumour-suppressor genes are marked in red. Non-essential genes are indicated in dark grey, and other genes are indicated in light grey. (B) Fold change graphs demonstrating the progressive depletion of pre-crRNAs targeting essential genes (red) from the *enAsCas12a*$^{KI/KI}$ MDFs over time, compared to other non-essential genes (black). (C-E) GSEA of Menuetto library MDF screen data at T1 (C), T2 (D), and T3 (E), each relative to T0, displaying the target gene classifications of pre-crRNAs depleted over the course of the experiment. Complete MDF-based drop-out screen analyses can be found in Supplementary File 3.

Supplementary Figure 8

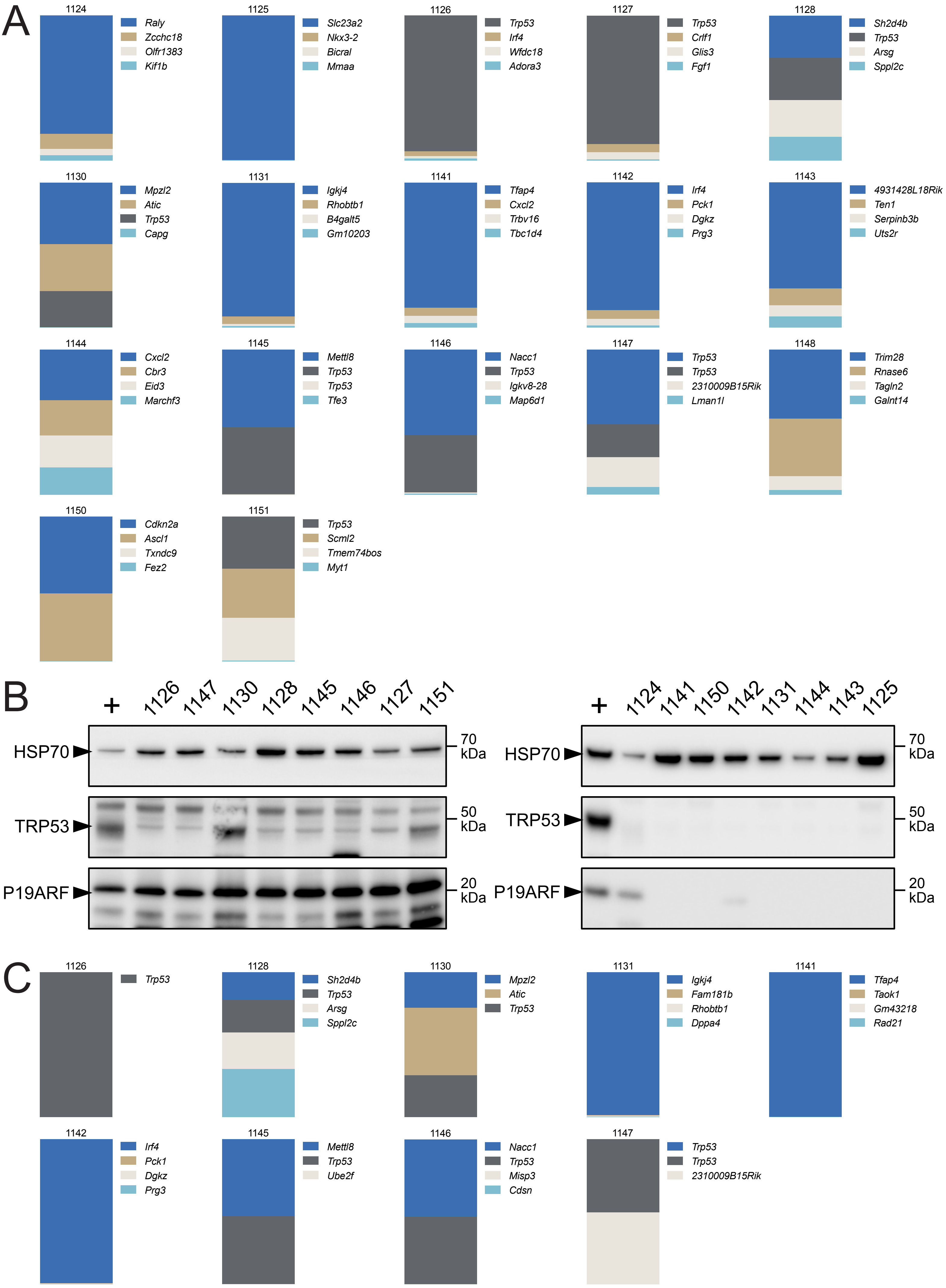

Supplementary Figure 8. Additional *in vivo* lymphoma Menuetto screening data. (A) Graphs showcasing the proportions of the top 4 represented pre-crRNAs in the tumour tissue of Menuetto library screen mice. (B) Western blots demonstrating TRP53 loss/mutation in tissues from each of the mice with enrichment of *Trp53*-targeting pre-crRNA, as evidenced by TRP53 stabilisation and P19ARF expression (left panel). Those mice without *Trp53* pre-crRNA enrichment appeared to have relatively normal TRP53 and P19ARF expression/signal. The left-most lane in each blot is a positive control sample derived from a spontaneously *Trp53*-mutant *Eμ-Myc* lymphoma. (C) Graphs showcasing the proportions of the top 4 represented pre-crRNAs in the cell lines that were able to be derived from the Menuetto screen library mice. Across all sequencing in A and C, read counts range from ~30,000-180,000, with average mapping percentages of ~88%. Source data are provided as a Source Data file.

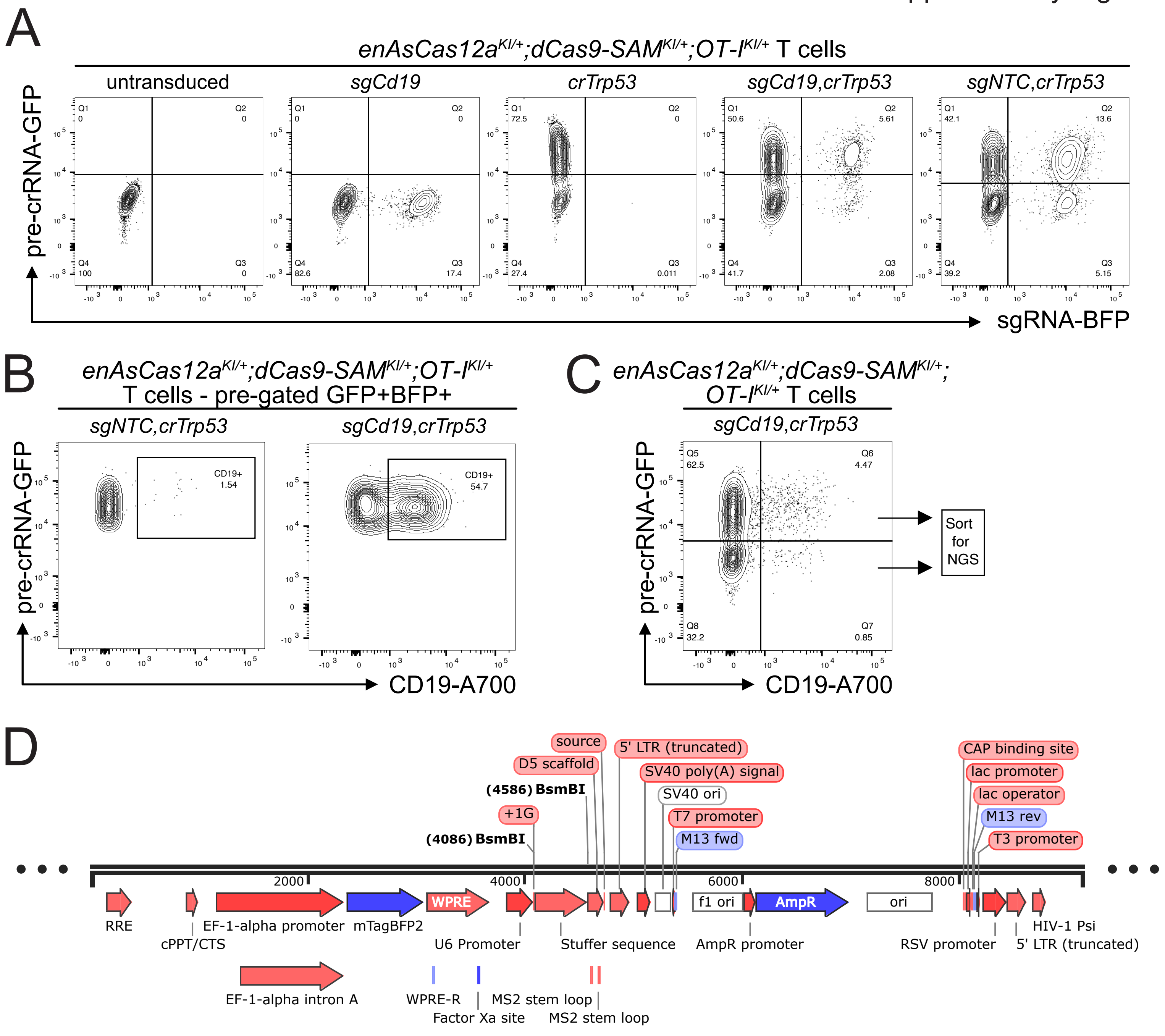

WEHI_9.2_pLKO_GNE_hU6_D5_TagBFP2
9137 bp (final length depends on sgRNA length post-stuffer sequence removal)

Supplementary Figure 9. Flow cytometry of multiplexed *enAsCas12a$^{KI/+}$;dCas9-SAM$^{KI/+}$;OT-I$^{T/+}$* T cells. (A) Representative FACS plots of *enAsCas12a$^{KI/+}$;dCas9-SAM$^{KI/+}$;OT-I$^{T/+}$* T cells (n=3 total) transduced with different, separate sgRNA and/or crRNA expression constructs. From left to right: untransduced, *sgCd19* (BFP+), *crTrp53* (GFP+), *sgCd19*,*crTrp53*, and *sgNTC*,*crTrp53*. (B) Representative FACS plots of *enAsCas12a$^{KI/+}$;dCas9-SAM$^{KI/+}$;OT-I$^{T/+}$* T cells (n=3 total) transduced with *crTrp53*, pre-gated on GFP+BFP+ cells. The cells additionally transduced with *sgNTC* show no CD19 expression (left), while those additionally transduced with a *sgCd19* show strong CD19 expression (right). (C) FACS plots of *enAsCas12a$^{KI/+}$;dCas9-SAM$^{KI/+}$;OT-I$^{T/+}$* T cells (n=1) doubly transduced with *crTrp53* and *sgCd19* constructs. CD19+ cells either GFP+ or GFP- (populations Q2, Q4) were sorted out for NGS to determine *Trp53* editing efficiency. (D) Vector map of the WEHI-9 vector, into which the *sgCd19* and *sgNTC* sequences were cloned. Abbreviations: crRNA = CRISPR RNA.

Supplementary Figure 10

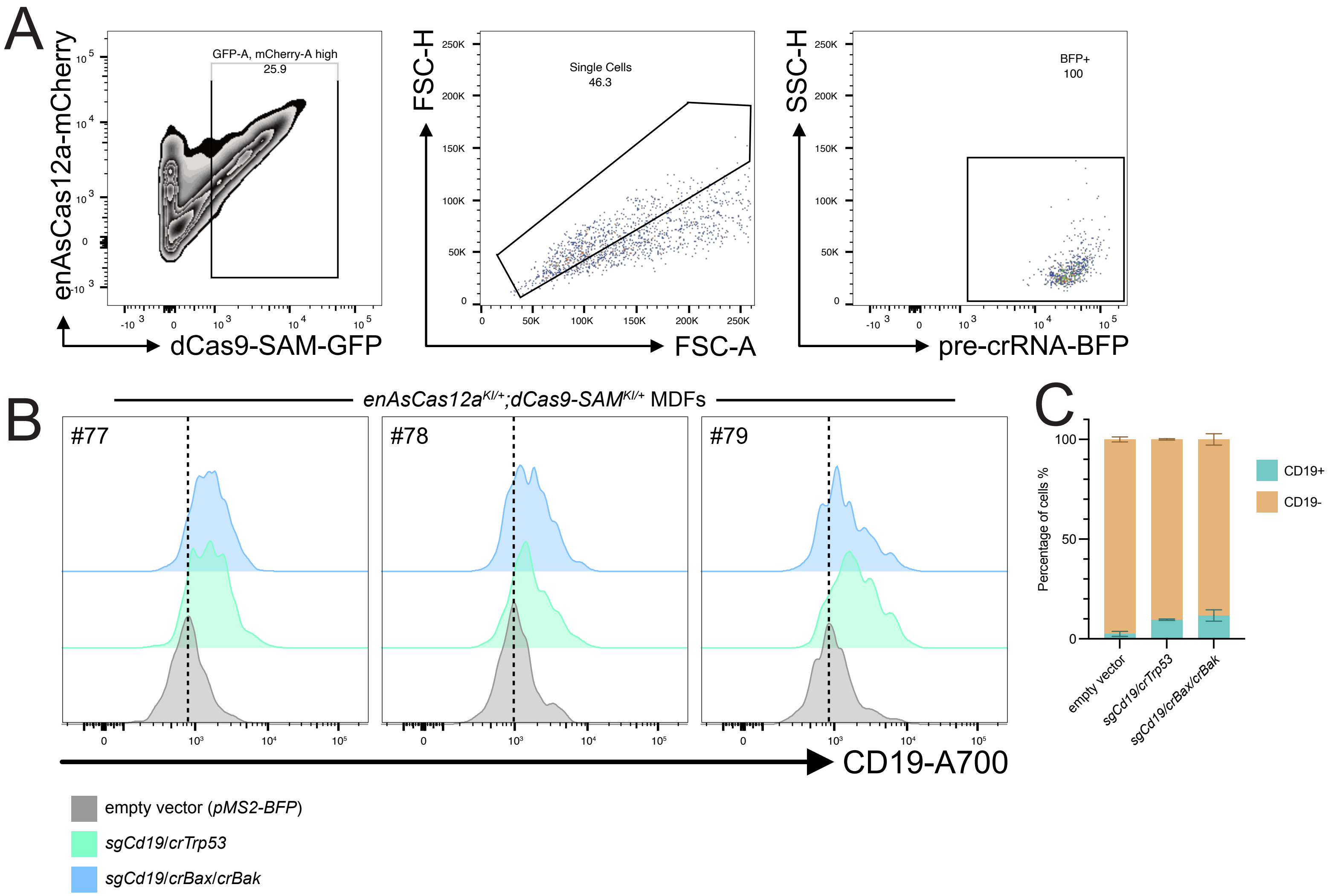

Supplementary Figure 10. Flow cytometry of multiplexed *enAsCas12a$^{KI/+}$;dCas9-SAM$^{KI/+}$* MDFs. (A) Representative (MDF line #77) gating strategy for the multiplexed *enAsCas12a$^{KI/+}$;dCas9$^{KI/+}$* MDFs (n=3). Cells were gated first on those with the strongest levels of mCherry and GFP expression (from enAsCas12a and dCas9-SAM expression, respectively), and lastly on BFP expression (from the pre-crRNA/sgRNA expression vector). (B) Histograms demonstrating CD19 upregulation in the multiplexed *enAsCas12a$^{KI/+}$;dCas9$^{KI/+}$* MDFs, transduced with a single vector co-expressing *sgCd19* and either *crTrp53* or *crBax/crBak* (n=3 independent transductions). Cells were gated on mCherry, GFP, and BFP expression. CD19 expression for the empty vector transduced MDFs are shown in grey, *crTrp53* in cyan, and *crBax/crBak* in blue. (C) Proportions of CD19+ cells sorted from *enAsCas12a$^{KI/+}$;dCas9$^{KI/+}$* MDFs transduced with expression vectors for *sgCd19/crTrp53* or *sgCd19/crBax/crBak* (or empty vector) (n=3 each). In each graph, the means are plotted, and the error bars represent SD. Statistical analyses of C can be found in Supplementary File 4. Source data are provided as a Source Data file. Abbreviations: crRNA = CRISPR RNA.

Supplementary Table 1. crRNA sequences. Sequences for each crRNA used in this study. Note that any lowercase letters refer to direct repeat sequences present in pre-crRNA arrays.

**crRNA Target**	**Sequence (5'-3')**
NTC	GGGCTCCGGCTCCTCAGAGAGCC
Trp53	AAGGCCCAAGTGAAGCCCTCCGA
Bim/Bcl2l11 – ex2	CTTGCAGAAAAAAAGACCAAATG
Bim/Bcl2l11 – ex3	GTTGAACTCGTCTCCGATCCGCC
Trp53/Bim/Puma/Noxa -targeting 4-tandem-guides array	aatttctactcttgtagatAAGGCCCAAGTGAAGCCCT<br>CCGAtaatttctactgtcgtagatCTTGCAGAAAAAAAGACCAAAT<br>GtaatttctactatcgtagatCCCTCACCCAGGTCCTCAGCCCtaaa<br>tttctactctagtagatAGAGCTACCACCTGAGTTCGCAG
Bax/Bak array	aatttctactcttgtagatATCCAGGATCGAGCAGGGAGGATtaattt<br>ctactgtcgtagatCCTCCACCAGCAGGAACAGGAGAtaatttctac<br>tttcgtagat
Trp53/Bim array	aatttctactcttgtagatAAGGCCCAAGTGAAGCCCTCCGAtaattt<br>ctactgtcgtagatGTTGAACTCGTCTCCGATCCGCCtaatttctact<br>ttcgtagat
Cd9/Cd81 array	aatttctactcttgtagatTCTCGGACACCTGCCCCAAGAAAtaatttc<br>tactgtcgtagatCGAAGCCCCAGATGCCTGCAGCCtaatttctactt<br>tcgtagat

Supplementary Table 2. sgRNA sequences. Sequences for each dCas9-SAM-compatible sgRNA used in this study.

**crRNA/sgRNA Target**	**Sequence (5'-3')**
NTC	GAAGGCCGCGTATCGTTGG
Cd19	TCCCTAAGTGCTGGGTGACAGGGA
Myc	TAGTGGGGAGCCGGGGAAAG

Supplementary Table 3. Overhang primers for NGS. Primers used to amplify gene regions and determine knockout efficacy via NGS. Note, the capitalised letters represent the gene targeting sequence, while the lowercase letters represent the overhang sequence.

**Target Gene**	**Sequence (5'-3')**
Trp53	FWD: gtgacctatgaactcaggagtcTTCTTTGTCCCATCCACAGC<br>REV: ctgagacttgcacatcgcagcCCACTCACCGTGCACATAAC
Bim/Bcl2l11 – ex2	FWD: gtgacctatgaactcaggagtcTGCTGAAGATAATCGTTTTGTTG<br>REV: ctgagacttgcacatcgcagcTGGGGATCTGGTAGCAAAAG
Bim/Bcl2l11 – ex3	FWD: gtgacctatgaactcaggagtcCCAGGGAGCTTCCAGAAGACC<br>REV: ctgagacttgcacatcgcagcGCCAAACCCTTAGATGCACCA
Puma/Bbc3 – ex3	FWD: gtgacctatgaactcaggagtcTAGGGCCTGTGGGAAAGAG<br>REV: ctgagacttgcacatcgcagcCAGCAGCTGCAGCACATC
Noxa/Pmaip1 – exon 2	FWD: gtgacctatgaactcaggagtcAAGCATCCGAGGATGTGC<br>REV: ctgagacttgcacatcgcagcACGAGCTCCAACGGACTAAG
Bax	FWD: gtgacctatgaactcaggagtcAACACCGCTTGGTTCTCAAC<br>REV: ctgagacttgcacatcgcagcGATGCGGCTCAAGGGTAAG
Bak	FWD: gtgacctatgaactcaggagtcATCTGCAGAACAGCAGGTTG<br>REV: ctgagacttgcacatcgcagcTCGTAGCGCCGGTTAATATC
Cd9	FWD: gtgacctatgaactcaggagtcATCCTCATCCCTGCCTTCTC<br>REV: ctgagacttgcacatcgcagcTGAAGGAGAACGGACTGACC
Cd81	FWD: gtgacctatgaactcaggagtcCTTGCTGGTCAAGGCTCTG<br>REV: ctgagacttgcacatcgcagcGCCCAAGAGCACTCAACTTC

Supplementary Table 4. Indexing primers used in NGS of non-screen samples. Sequences represent the overhanging index sequences only, and not the entire primer sequence. The remaining sequence for each primer is complementary to the (lowercase) overhang sequences shown in Table S3.

**Index name (forward)**	**Index sequence (5'-3')**	**Index name (reverse)**	**Index sequence (5'-3')**
Fwd_1	TAGATCGC	Rev_01	TAAGGCGA
Fwd_3	TATCCTCT	Rev_02	CGTACTAG
Fwd_4	AGAGTAGA	Rev_03	AGGCAGA
Fwd_5	GTAAGGAG	Rev_05	AGGCAGAA
Fwd_6	ACTGCATA	Rev_6	TAGGCATG
Fwd_7	AAGGAGTA	Rev_07	CTCTCTAC
Fwd_10	GAGCTGAA	Rev_08	CAGAGAGG
Fwd_11	GCGAGTAA	Rev_09	GCTACGCT
Fwd_12	TGAAGAGA	Rev_10	CGAGGCTG
Fwd_13	TGGTGGTA	Rev_11	AAGAGGCA
Fwd_14	TTCACGCA	Rev_12	GTAGAGGA
Fwd_15	AGCACCTC	Rev_13	ATGCCTAA
Fwd_16	CAAGGAGC	Rev_14	ACGCTCGA
Fwd_17	ATTGGCTC	Rev_15	AGTCACTA
Fwd_18	CACCTTAC	Rev_16	ATCCTGTA
Fwd_19	CTAAGGTC	Rev_17	CGCATACA
Fwd_20	GAACAGGC		
Fwd_21	CCGTGAGA		
Fwd_22	CCTCCTGA		
Fwd_23	CGAACTTA		
Fwd_24	CGACTGGA		

Supplementary Table 5. Overhang primers used in NGS to assess off-target editing. For each on-target gene-editing performed with individual crRNAs or the 4-tandem guide array, off-target activity was examined via NGS. Note that no off-targets were identified for *crBim* (ex3), and 5 of the 17 potential off-targets for the *crBim* (ex2) were tested. Further note that the crRNA sequences for each target are given in Table S1, and the NGS indexing primers used are given in Table S3.

crRNA (sequences in Table S1)	Potential off-target DNA sequence	Forward primer (5'-3')	Reverse primer (5'-3')	Chromosome	Chromosome Position
Trp53	TTTGAAGGCCCAAGTGAAGCCCTCCAA	CAGGCTTATGGAAACTACTTCCTC	CCAAGTCTGTTACTGTACTCTCC	17	54419829
Bim/Bcl2l11 – ex2 (1)	TTTTCTAGCAGAGAAAAAAACCAAATG	ACTGAGAGACATAATCAGCTAGG	GCCACAGACAGCATCCTGTGT	5	39423237
Bim/Bcl2l11 – ex2 (2)	TTTACTTCCAGAAAAAAAGAGTAAAGG	GGTTTCTCACACAATACAGCACAC	TACCCACTCCTTCCCCTAACA	15	15013528
Bim/Bcl2l11 – ex2 (3)	TTTCTTTGAAAAAAAAAAAACCAAATG	AGTTGTCACCTCTTGACTCAATC	TCCCTCTCCACCATTCACATTC	5	28650388
Bim/Bcl2l11 – ex2 (4)	TTTGCTTGCAGTAAGAAAGACAAAATC	ACGAGTCCAAAGGGAAATCTAGC	ACCTGGTGCCTATCTTGCTATG	2	159489954
Bim/Bcl2l11 – ex2 (5)	TTTTCTTCCAGAAAAACCTACCAAATG	ATTGTCACATCCAGCTCTTGC	AAACATAGGGTTATGGCACAGAC	12	89236623
Puma/Bbc3 (1)	TTTCCCCTCtCCCAGcTCCcCAGCCCT	AGCATTCTCTCTCTCCCTTTCTGTC	GATGAAAGGTGTACCCAAGCTGT	17	65224559
Puma/Bbc3 (2)	TTTACCCTTTCCCAGGTCCTCACTCCT	CACAGGCTGAGAGAAGAGATTCTAG	CAAAGGTTACAGGAGATGGGAGT	16	64241748
Noxa/Pmaip1 (1)	TTTGAGTGCTAGCACCTGAGTTCCAAG	AGAATGATAAACAACACGATGCAGC	CATCAGTGTGCCCTTTTTCTTCTCT	2	72456295
Noxa/Pmaip1 (2)	TTTGAAAGCTCCCACCTTAGTTGGCAG	ATCAGTAACACACACACACACTCAC	CTGGAGCATGAGTTTCAACAAGTT	12	67507415

Supplementary Table 6. Indexing primers used in NGS of whole-genome screen samples. Entire sequences for the forward and reverse primers used are given. Note, the capitalised letters represent the gene targeting sequence, while the lowercase letters represent the overhanging index sequence.

**Forward index IDs and sequences (5'-3')**	
35	aatgatacggcgaccaccgagatctacactctttccctacacgacgctcttccgatctgtctgtcaTCTTGTGGAAAGGACGAGGTACCG
36	aatgatacggcgaccaccgagatctacactctttccctacacgacgctcttccgatcttaggatgatgTCTTGTGGAAAGGACGAGGTACCG
117	aatgatacggcgaccaccgagatctacactctttccctacacgacgctcttccgatctgattacttTCTTGTGGAAAGGACGAGGTACCG
118	aatgatacggcgaccaccgagatctacactctttccctacacgacgctcttccgatcttcaattgcatTCTTGTGGAAAGGACGAGGTACCG
119	aatgatacggcgaccaccgagatctacactctttccctacacgacgctcttccgatctaacacttactgTCTTGTGGAAAGGACGAGGTACCG
120	aatgatacggcgaccaccgagatctacactctttccctacacgacgctcttccgatctccctactccTCTTGTGGAAAGGACGAGGTACCG
**Reverse index IDs and sequences (5'-3')**	
47	caagcagaagacggcatacgagatcggtctcggcattcctgctgaaccgctcttccgatctaagacggaTCTACTATTCTTTCCCCTGCACTGT
48	caagcagaagacggcatacgagatcggtctcggcattcctgctgaaccgctcttccgatctaaggtacaaTCTACTATTCTTTCCCCTGCACTGT
49	caagcagaagacggcatacgagatcggtctcggcattcctgctgaaccgctcttccgatcttagcgagtgcTCTACTATTCTTTCCCCTGCACTGT
50	caagcagaagacggcatacgagatcggtctcggcattcctgctgaaccgctcttccgatctgtagctcctacTCTACTATTCTTTCCCCTGCACTGT
51	caagcagaagacggcatacgagatcggtctcggcattcctgctgaaccgctcttccgatcttactacgcctTCTACTATTCTTTCCCCTGCACTGT
52	caagcagaagacggcatacgagatcggtctcggcattcctgctgaaccgctcttccgatctaggctccgTCTACTATTCTTTCCCCTGCACTGT
59	caagcagaagacggcatacgagatcggtctcggcattcctgctgaaccgctcttccgatctagatcgcaTCTACTATTCTTTCCCCTGCACTGT
60	caagcagaagacggcatacgagatcggtctcggcattcctgctgaaccgctcttccgatctagcaggaatTCTACTATTCTTTCCCCTGCACTGT
61	caagcagaagacggcatacgagatcggtctcggcattcctgctgaaccgctcttccgatctgcagcgtaagTCTACTATTCTTTCCCCTGCACTGT
62	caagcagaagacggcatacgagatcggtctcggcattcctgctgaaccgctcttccgatctctgcgcatcagTCTACTATTCTTTCCCCTGCACTGT
63	caagcagaagacggcatacgagatcggtctcggcattcctgctgaaccgctcttccgatctgagcgctaTCTACTATTCTTTCCCCTGCACTGT
64	caagcagaagacggcatacgagatcggtctcggcattcctgctgaaccgctcttccgatctcgctcagtgTCTACTATTCTTTCCCCTGCACTGT

Source Data - Supplementary Figure gels/blots

Supplementary Figure 1A

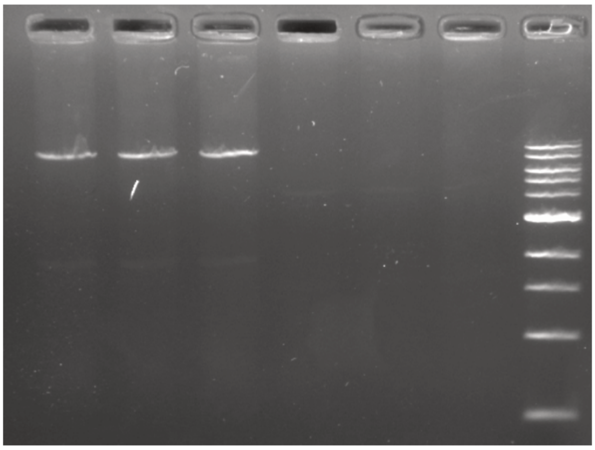

Supplementary Figure 3B

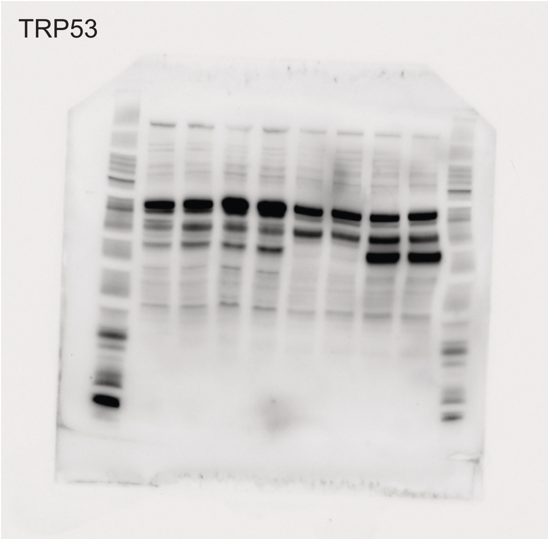

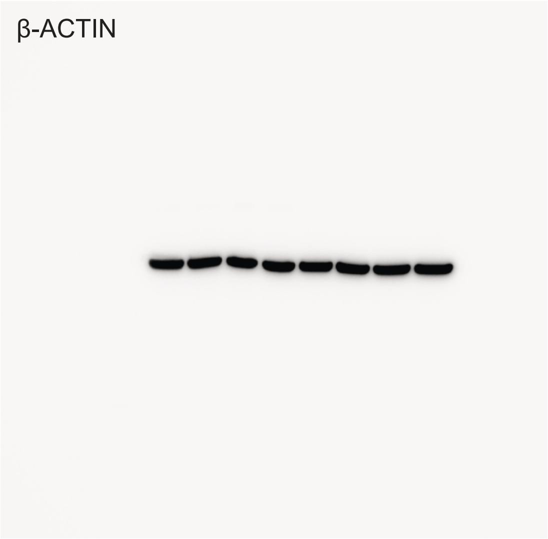

Supplementary Figure 8B

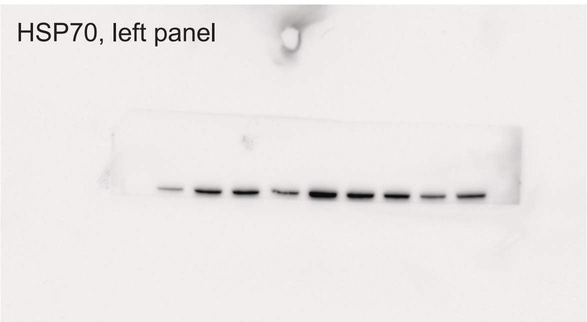

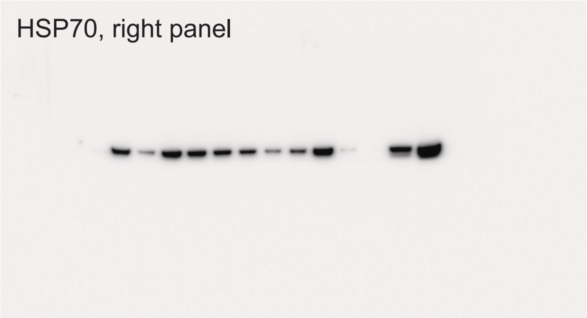

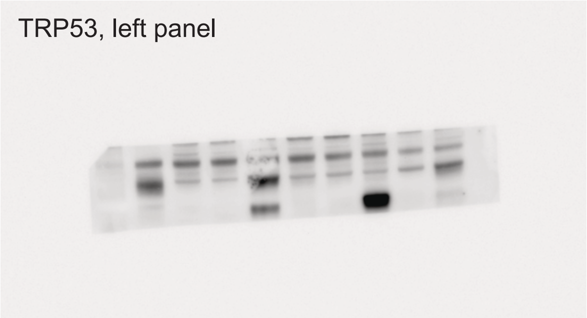

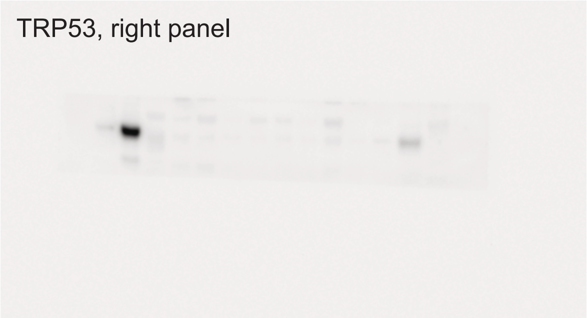

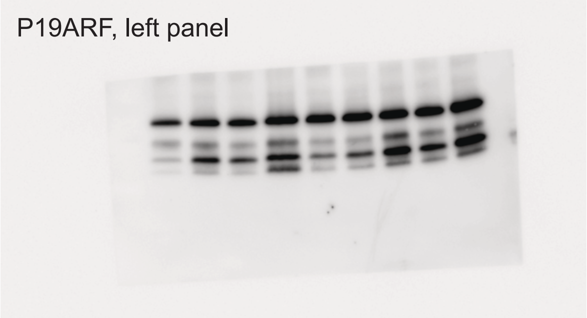

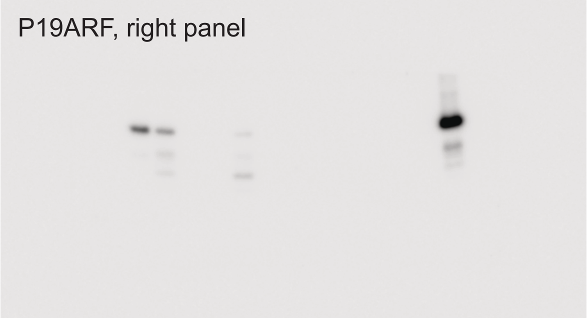

Supplement: Supplementary file 1 — Supplementary Information [file 41467_2025_56282_MOESM1_ESM.pdf]
