# Supplementary material for: Advancing the genetic engineering toolbox by combining AsCas12a knock-in mice with ultra-compact screening
Source: Nat Commun. 2025 Jan 30;16:974. doi: 10.1038/s41467-025-56282-2 (PMC11782673; doi:10.1038/s41467-025-56282-2)
Supplement: Supplementary file 2 — Description of Additional Supplementary Files [file 41467_2025_56282_MOESM2_ESM.pdf]

Supplementary File Information

File Name: Supplementary Data 1 - Cas12a crRNA expression libraries
Description: Complete information for each of the Menuetto and Scherzo Cas12a pre-crRNA libraries, including gene-specific pre-crRNA sequences.

File Name: Supplementary Data 2 - Lymphoma-based Menuetto/Scherzo screen analyses
Description: DMSO, nutlin-3a, or S63845 vs input comparisons for each of the whole-genome Cas12a screens conducted in *Eμ-Myc*$^{T/+}$*;enAsCas12a*$^{KI/+}$ lymphoma cells. For the Menuetto (dual) library, there are gRNA and gene level comparisons. For the Scherzo (quad) library, only gRNA level comparisons are shown, as this is indistinguishable from the gene level due to the design of the library. All comparisons are sorted by FDR.

File Name: Supplementary Data 3 - Immortalised MDF drop-out screen analyses
Description: T0 vs T1/T2/T3 analyses for the Menuetto library drop-out screen conducted in *enAsCas12a*$^{KI/KI}$ immortalised MDFs. Analyses of both the guide/pre-crRNAs and their target genes are included, with all comparisons sorted by FDR.

File Name: Supplementary Data 4 - Statistical analyses
Description: Analyses were performed for each NGS experiment using a two-way ANOVA with Šídák's multiple comparisons test. Only relevant comparisons are tabulated.
