# Supplementary material for: Advancing the genetic engineering toolbox by combining AsCas12a knock-in mice with ultra-compact screening
Source: Nat Commun. 2025 Jan 30;16:974. doi: 10.1038/s41467-025-56282-2 (PMC11782673; doi:10.1038/s41467-025-56282-2)
Supplement: Supplementary file 6 — Supplementary Data 4 [file 41467_2025_56282_MOESM6_ESM.docx]

**Supplementary File 4 – Statistical analyses.**

**Fig. 1B: MDFs – constitutive individual pre-crRNAs.** NGS analysis using a two-way ANOVA with Šídák's multiple comparisons test. Only the p-values and significance results for relevant comparisons are shown. *=p<0.05, ****=<0.0001.

| **Empty control vs. individual pre-crRNA** | | | | |
| --- | --- | --- | --- | --- |
| **Target gene** | **Cell line genotype** | **frameshift** | **in-frame indel** | **wild-type** |
| *Trp53* | *enAsCas12a^KI/KI^* | <0.0001, **** | <0.0001, **** | <0.0001, **** |
| *Bim/Bcl2l11* (ex2) | *enAsCas12a^KI/KI^* | <0.0001, **** | 0.0159, * | <0.0001, **** |
| *Bim/Bcl2l11* (ex3) | *enAsCas12a^KI/KI^* | <0.0001, **** | 0.0223, * | <0.0001, **** |

**Fig. 1C: MDFs – constitutive 4-tandem-guides.** NGS analysis using a two-way ANOVA with Šídák's multiple comparisons test. Only the p-values and significance results for relevant comparisons are shown. ****=<0.0001.

| **Empty control vs 4-tandem-guides** | | | | |
| --- | --- | --- | --- | --- |
| **Target gene** | **Cell line genotype** | **frameshift** | **in-frame indel** | **wild-type** |
| *Trp53* | *enAsCas12a^KI/KI^* | <0.0001, **** | <0.0001, **** | <0.0001, **** |
| *Bim/Bcl2l11* | *enAsCas12a^KI/KI^* | <0.0001, **** | <0.0001, **** | <0.0001, **** |
| *Puma/Bbc3* | *enAsCas12a^KI/KI^* | <0.0001, **** | <0.0001, **** | <0.0001, **** |
| *Noxa/Pmaip1* | *enAsCas12a^KI/KI^* | <0.0001, **** | <0.0001, **** | <0.0001, **** |

**Fig. 1H, I: Reconstitution experiment using homozygous/heterozygous Cas12a.** NGS analysis using a two-way ANOVA with Šídák's multiple comparisons test. Only the p-values and significance results for relevant comparisons are shown. **=p<0.01.

| **Heterozygous *enAsCas12a^KI/+^* vs. homozygous *enAsCas12a^KI/KI^*** | | | | |
| --- | --- | --- | --- | --- |
| **Target gene** | **Tissue** | **frameshift** | **in-frame indel** | **wild-type** |
| *Trp53* | Thymus | 0.0016, ** | 0.9998, ns | 0.0041, ** |
|  | Spleen | 0.8684, ns | >0.9999, ns | 0.7634, ns |
| *Bim/Bcl2l11* | Thymus | 0.2762, ns | 0.9943, ns | 0.6420, ns |
|  | Spleen | 0.9313, ns | 0.9966, ns | 0.9989, ns |

**Fig. 4G: BFP/CD19 sorting of *enAsCas12a^KI/+^;dCas9^KI/+^* MDFs with *sgCd19*/*crTrp53*, *sgCd19*/*crBax*/*crBak*.** NGS analysis using a two-way ANOVA with Šídák's multiple comparisons test. Only the p-values and significance results for relevant comparisons are shown. **=p<0.01, ****=<0.0001.

| **BFP+ cells vs. BFP+CD19+ cells** | | | |
| --- | --- | --- | --- |
| **Target gene** | **frameshift** | **in-frame indel** | **wild-type** |
| *Trp53* | <0.0001, **** | 0.0024, ** | <0.0001, **** |
| *Bax* | <0.0001, **** | 0.5788, ns | <0.0001, **** |
| *Bak* | <0.0001, **** | 0.0057, ** | <0.0001, **** |

**Fig. S1G. qRT-PCR of *enAsCas12a* expression between heterozygous and homozygous tissues.** *enAsCas12a* expression analysis using a two-way ANOVA with Tukey’s multiple comparisons test. Each tissue was compared only to itself, and each p-value and significance result is shown. *=p<0.05, **=p<0.01, ***=p<0.001, ****=<0.0001.

| **Wild-type (WT) vs. *enAsCas12a^KI/+^* (HET) vs. *enAsCas12a^KI/KI^* (HOM)** | | | |
| --- | --- | --- | --- |
| **Tissue** | **WT vs. HET** | **WT vs. HOM** | **HET vs. HOM** |
| Thymus | 0.1082, ns | <0.0001, **** | 0.0135, * |
| Spleen | 0.0060, ** | 0.0002, *** | 0.4578, ns |
| Kidney | 0.6030, ns | 0.0008, *** | 0.0121, * |
| Liver | 0.4815, ns | <0.0001, **** | <0.0001, **** |
| Brain | 0.8033 | 0.0002, *** | 0.0011, ** |
| Bone marrow | 0.0536, ns | <0.0001, **** | 0.0219, * |
| Heart | 0.7278, ns | <0.0001, **** | <0.0001, **** |

**Fig. S3A: iMDFs - constitutive individual pre-crRNAs.** NGS analysis using a two-way ANOVA with Šídák's multiple comparisons test. Only the p-values and significance results for relevant comparisons are shown. ****=<0.0001.

| **Empty control vs. individual pre-crRNA** | | | | |
| --- | --- | --- | --- | --- |
| **Target gene** | **Cell line genotype** | **frameshift** | **in-frame indel** | **wild-type** |
| *Trp53* | *enAsCas12a^KI/KI^* | <0.0001, **** | <0.0001, **** | <0.0001, **** |
| *Bim/Bcl2l11* | *enAsCas12a^KI/KI^* | <0.0001, **** | <0.0001, **** | <0.0001, **** |

**Fig. S3A: iMDFs - constitutive dual-pre-crRNAs.** NGS analysis using a two-way ANOVA with Šídák's multiple comparisons test. Only the p-values and significance results for relevant comparisons are shown. ****=<0.0001.

| **Empty control vs. dual pre-crRNAs** | | | | |
| --- | --- | --- | --- | --- |
| **Target gene** | **Dual pre-crRNAs** | **frameshift** | **in-frame indel** | **wild-type** |
| *Trp53* | *crTrp53/crBim* | <0.0001, **** | <0.0001, **** | <0.0001, **** |
| *Bim/Bcl2l11* |  | <0.0001, **** | <0.0001, **** | <0.0001, **** |
| *Cd9* | *crCd9/crCd81* | <0.0001, **** | <0.0001, **** | <0.0001, **** |
| *Cd81* |  | <0.0001, **** | <0.0001, **** | <0.0001, **** |

**Fig. S3C: *EμMyc^T/+^* lypmphoma cells – constitutive individual pre-crRNAs.** NGS analysis using a two-way ANOVA with Šídák's multiple comparisons test. Only the p-values and significance results for relevant comparisons are shown. *=p<0.05, ***=p<0.001.

| **Empty control vs. individual pre-crRNA** | | | | |
| --- | --- | --- | --- | --- |
| **Target gene** | **Cell line genotype** | **frameshift** | **in-frame indel** | **wild-type** |
| *Trp53* | *EμMyc^T/+^; enAsCas12a^KI/+^* | 0.0111, * | 0.3566, ns | 0.0007, *** |
| *Bim/Bcl2l11* (ex2) |  | 0.0666, ns | 0.9403, ns | 0.0255, * |
| *Bim/Bcl2l11* (ex3) |  | 0.1466, ns | 0.9212, ns | 0.0524, ns |

**Fig. S3D: *EμMyc^T/+^* lymphoma cells – constitutive 4-tandem-guides.** NGS analysis using a two-way ANOVA with Šídák's multiple comparisons test. Only the p-values and significance results for relevant comparisons are shown. *=p<0.05, **=p<0.01, ***=p<0.001, ****=<0.0001.

| **Empty control vs. 4-tandem-guides** | | | | |
| --- | --- | --- | --- | --- |
| **Target gene** | **Cell line genotype** | **frameshift** | **in-frame indel** | **wild-type** |
| *Trp53* | *EμMyc^T/+^; enAsCas12a^KI/+^* | <0.0001, **** | <0.0001, **** | <0.0001, **** |
| *Bim/Bcl2l11* |  | 0.0016, ** | 0.9727, ns | 0.0031, ** |
| *Puma/Bbc3* |  | 0.0053, ** | 0.3366, ns | 0.0003, *** |
| *Noxa/Pmaip1* |  | 0.0165, * | 0.9916, ns | 0.0102, * |

**Fig. S10C: CD19+/CD19- percentage in *enAsCas12a^KI/+^;dCas9^KI/+^* MDFs with empty control,  *sgCd19/crTrp53*, or *sgCd19/crBax/crBak*.** Cell proportions analysis using a two-way ANOVA with Šídák's multiple comparisons test. Only the p-values and significance results for relevant comparisons are shown. **=p<0.01, ***=p<0.001.

| **Comparison** | **P value and significance** |
| --- | --- |
| Empty control vs. *sgCd19/crTrp53* | 0.0013, ** |
| Empty control vs. *sgCd19/crBax/crBak* | 0.0001, *** |
| *sgCd19/crTrp53* vs. *sgCd19/crBax/crBak* | 0.4472, ns |
