# Supplementary material for: Advancing the genetic engineering toolbox by combining AsCas12a knock-in mice with ultra-compact screening
Source: Nat Commun. 2025 Jan 30;16:974. doi: 10.1038/s41467-025-56282-2 (PMC11782673; doi:10.1038/s41467-025-56282-2)
Supplement: Supplementary file 7 — Reporting Summary [file 41467_2025_56282_MOESM7_ESM.pdf]

nature portfolio

Corresponding author(s): Marco J. Herold

Last updated by author(s): Jan 4, 2025

Reporting Summary

Nature Portfolio wishes to improve the reproducibility of the work that we publish. This form provides structure for consistency and transparency in reporting. For further information on Nature Portfolio policies, see our Editorial Policies and the Editorial Policy Checklist.

Statistics

For all statistical analyses, confirm that the following items are present in the figure legend, table legend, main text, or Methods section.

n/a	Confirmed	
☐	☒	The exact sample size (n) for each experimental group/condition, given as a discrete number and unit of measurement
☐	☒	A statement on whether measurements were taken from distinct samples or whether the same sample was measured repeatedly
☐	☒	The statistical test(s) used AND whether they are one- or two-sided<br>*Only common tests should be described solely by name; describe more complex techniques in the Methods section.*
☒	☐	A description of all covariates tested
☐	☒	A description of any assumptions or corrections, such as tests of normality and adjustment for multiple comparisons
☐	☒	A full description of the statistical parameters including central tendency (e.g. means) or other basic estimates (e.g. regression coefficient) AND variation (e.g. standard deviation) or associated estimates of uncertainty (e.g. confidence intervals)
☒	☐	For null hypothesis testing, the test statistic (e.g. F, t, r) with confidence intervals, effect sizes, degrees of freedom and P value noted<br>*Give P values as exact values whenever suitable.*
☒	☐	For Bayesian analysis, information on the choice of priors and Markov chain Monte Carlo settings
☒	☐	For hierarchical and complex designs, identification of the appropriate level for tests and full reporting of outcomes
☐	☒	Estimates of effect sizes (e.g. Cohen's d, Pearson's r), indicating how they were calculated

Our web collection on statistics for biologists contains articles on many of the points above.

Software and code

Policy information about availability of computer code

Data collection	Flow cytometry data was captured using FACSDiva software (BD Biosciences, v8.0+).<br>Gel and blot images were captured using Image Lab software (Bio-Rad).<br>qRT-PCR data was captured using QuantStudio 12K Flex Real-Time PCR software.
Data analysis	Flow cytometry data was analysed using FlowJo (BD Biosciences, v8.0+).<br>General data analysis, including graph creation and statistical testing, were performed using Prism (v9+, GraphPad).<br>Screening data was analysed (in part) using MAGeCK (v0.5+).<br>Statistical analyses of the dual and quad screens were performed using the limma package (v3.6.1) within the R software (v4.4.1).

For manuscripts utilizing custom algorithms or software that are central to the research but not yet described in published literature, software must be made available to editors and reviewers. We strongly encourage code deposition in a community repository (e.g. GitHub). See the Nature Portfolio guidelines for submitting code & software for further information.

Data

Policy information about availability of data

All manuscripts must include a data availability statement. This statement should provide the following information, where applicable:

- Accession codes, unique identifiers, or web links for publicly available datasets
- A description of any restrictions on data availability
- For clinical datasets or third party data, please ensure that the statement adheres to our policy

Library and screening data is available in Supplementary Material. Source data are provided as a Source Data file. Raw sequencing data is available on the NCBI GEO repository (GSE285778). We believe all necessary data has been made available, but if any additional data is desired by a reader, it will be made available as quickly as possible upon request to the corresponding author, with no conditions made on access.

Research involving human participants, their data, or biological material

Policy information about studies with human participants or human data. See also policy information about sex, gender (identity/presentation), and sexual orientation and race, ethnicity and racism.

Reporting on sex and gender	Not applicable.
Reporting on race, ethnicity, or other socially relevant groupings	Not applicable.
Population characteristics	Not applicable.
Recruitment	Not applicable.
Ethics oversight	Not applicable.

Note that full information on the approval of the study protocol must also be provided in the manuscript.

Field-specific reporting

Please select the one below that is the best fit for your research. If you are not sure, read the appropriate sections before making your selection.

☒ Life sciences ☐ Behavioural & social sciences ☐ Ecological, evolutionary & environmental sciences

For a reference copy of the document with all sections, see nature.com/documents/nr-reporting-summary-flat.pdf

Life sciences study design

All studies must disclose on these points even when the disclosure is negative.

Sample size	Sample size calculations were not performed. Almost all in vitro assays were performed with n=3 biological replicates, to ensure statistical inferences could be drawn. The few exceptions were due to there being a very low degree of variability between samples (where n=2), or the experiment being performed only to obtain data that would inform additional assays (where n=1). The latter, for example, occurred with the data in Fig. S6A,B. Mouse cohorts were chosen as being sufficient to observe desired effects (for controls) and obtain statistically reliable data, while also working in concordance with the 3Rs, and keeping samle sizes as low as possible to also demonstrate the enAsCas12a model can be used to potentially reduce the number of mice required for in vivo screening.
Data exclusions	Some mice were excluded from survival curves (and downstream analyses) when they were determined to have died from a cause other than the disease being modelled (i.e. lymphma). This exclusion criteria is standard for these types of experiments, and is indicated as having occurred in the relevant text section.
Replication	All replciate numbers are indicated in the manuscript. All experiments were performed 2-3 times, with the exception of data found in Fig. 4C. However, this data is sequencing data that appears largely consistent with similar data throughout the manuscript, and so it is extremely unlikely that additional replicates will alter this outcome.
Randomization	All mice used in all experiments were randomly assigned to each group, after accounting for genotype, and the matching of groups based on age and gender.
Blinding	Highly trained animal technicians were solely responsible for calling ethical endpoint status of mice, and were blinded to the nature of each experiment.

Reporting for specific materials, systems and methods

We require information from authors about some types of materials, experimental systems and methods used in many studies. Here, indicate whether each material, system or method listed is relevant to your study. If you are not sure if a list item applies to your research, read the appropriate section before selecting a response.

Materials & experimental systems

n/a	Involved in the study	
☐	☒	Antibodies
☐	☒	Eukaryotic cell lines
☒	☐	Palaeontology and archaeology
☐	☒	Animals and other organisms
☒	☐	Clinical data
☒	☐	Dual use research of concern
☒	☐	Plants

Methods

n/a	Involved in the study	
☒	☐	ChIP-seq
☐	☒	Flow cytometry
☒	☐	MRI-based neuroimaging

Antibodies

Antibodies used

FACS antibodies used were:
B220 (RA3-6B2-BV605; 1:200; BioLegend #103244)
TCRβ (H57-597-PE-Cy7; 1:400; BioLegend #109222)
Mac1 (M1/70-APC-Cy7; 1:400; BD Biosciences #557657)
Gr1 (RB68C5-Alexa Fluor 700; 1:400; made in house)
IgM (5-1-FITC or 5-1-PE; 1:400; made in house)
IgD (11-26c.2a-BV510; 1:400; BD Biosciences #563110)
CD4 (GK1.5-PerCP-Cy5.5; 1:800; BioLegend #100434)
CD8 (53.6.7-Alexa Fluor 647; 1:400; made in house)
CD19 (ID3-A700; 1:400; made in house)
Ly5.1 (A20.1-PE; 1:400; made in-house)
Ly5.2 (S450-Alexa Fluor 700; 1:400; made in-house)

Western blotting antiboides used were:
P53 (1:2000; Novocastra #NCL-p53-CM5p)
B -ACTIN (1:2000; Sigma #A2228)
BAX (1:2000; Sigma Aldrich #B9054)
BAK (1:2000; Sigma Aldrich #5897)
HSP70 (1:10,000; gift from Dr R Anderson, ONJCRI)

Validation

Vaildation of commercially obtainable antibodies is available on the manufacturer's website.
Antibodies manufactured in-house (or by other labs) have a long history of use in a range of published manuscripts, and have been validated numerous times as binding to proteins of the appropriate size and/or intensity (in cells manipulated for the levels of these protein targets), or proteisn that are present in the appropriate cell type (as evidenced by combinatorial cell surface markers staining).

Eukaryotic cell lines

Policy information about cell lines and Sex and Gender in Research

Cell line source(s)

HEK293T cells were purchased from the ATCC (#CRL216; RRID: CVCL_0063).
All other cell lines were generated as part of this study, as described within the manuscript.

Authentication

HEK293T cells were authenticated via morphological and functional observations, as well as via protocols obtained from the ATCC.
Lymphoma cell lines were valdiated via immunophenotyping of the tissues from which they derive, and testing for spontaneous Trp53 mutation.
Other cell lines were not validated, beyong morphological observations.

Mycoplasma contamination

Cell lines were routinely determined to be negative for Mycoplasma infection using a MycoALert detection kit (Lonza #LT07-118).

Commonly misidentified lines
(See ICLAC register)

No commonly misidentified cell lines were used.

Animals and other research organisms

Policy information about studies involving animals; ARRIVE guidelines recommended for reporting animal research, and Sex and Gender in Research

Laboratory animals

All mice used in this study are of the sepcies Mus musculus, and were maintained on a C57BL/6 background.

Laboratory animals	enAsCas12a mice were developed as part of this study.<br>All mice used in experiments were age-matched. Generally, mice used in experiments were between 6-12 weeks old.
Wild animals	No wild animals were used in this study.
Reporting on sex	No sex-based analyses were performed as part of this study, as they are not bleived to be relevant.
Field-collected samples	No field collected samples were used in this study.
Ethics oversight	Care and husbandry of experimental mice was performed according to the guidelines established by both The Walter and Eliza Hall Institute Animal Ethics Committee and the Austin Health Animal Ethics Committee.

Note that full information on the approval of the study protocol must also be provided in the manuscript.

Plants

Seed stocks	*Report on the source of all seed stocks or other plant material used. If applicable, state the seed stock centre and catalogue number. If plant specimens were collected from the field, describe the collection location, date and sampling procedures.*
Novel plant genotypes	*Describe the methods by which all novel plant genotypes were produced. This includes those generated by transgenic approaches, gene editing, chemical/radiation-based mutagenesis and hybridization. For transgenic lines, describe the transformation method, the number of independent lines analyzed and the generation upon which experiments were performed. For gene-edited lines, describe the editor used, the endogenous sequence targeted for editing, the targeting guide RNA sequence (if applicable) and how the editor was applied.*
Authentication	*Describe any authentication procedures for each seed stock used or novel genotype generated. Describe any experiments used to assess the effect of a mutation and, where applicable, how potential secondary effects (e.g. second site T-DNA insertions, mosiacism, off-target gene editing) were examined.*

Flow Cytometry

Plots

Confirm that:

☒ The axis labels state the marker and fluorochrome used (e.g. CD4-FITC).

☒ The axis scales are clearly visible. Include numbers along axes only for bottom left plot of group (a 'group' is an analysis of identical markers).

☒ All plots are contour plots with outliers or pseudocolor plots.

☒ A numerical value for number of cells or percentage (with statistics) is provided.

Methodology

Sample preparation	To perform FACS on haematopoietic cells/tissues (e.g. peripheral blood samples, bone marrow, thymi, spleens, and lymph nodes), cells were harvested from the mice and processed into single cell suspensions (where necessary). Red blood cells were removed (where necessary – e.g. peripheral blood, spleen) by addition of red cell lysis buffer (made in-house: ammonium chloride (156 mM), sodium bicarbonate (11.9 mM), EDTA (0.097mM)) before the cells were washed twice with 1 PBS (Gibco #14190144), centrifuged, and then resuspended in FACS buffer (1 PBS, EDTA (5µM) (Sigma-Aldrich #E8008), 5% FBS (Sigma-Aldrich #12007C)).<br>To perform FACS on adherent cells (e.g. MDFs), cells were first detached from their plates using cell scrapers (Corning, #3010), placed into FACS buffer, filtered, washed and centrifuged using 1 PBS, before resuspension in FACS buffer.<br>To perform FACS on suspension cells, cells were first filtered, washed and centrifuged using 1 PBS, before resuspension in FACS buffer.<br>As needed, cells were counted using a TC20 Automated Cell Counter (BioRad). Once prepared, single-cell suspensions resuspended in a cocktail of FACS buffer with anti-FCR (made in house; 1:10) and the fluorochrome-conjugated antibodies against proteins of interest.<br>Cells were incubated in the antibody cocktail, on ice, for at least 25 m, then washed twice by centrifugation with 1 PBS, and resuspended in FACS buffer for analysis. At the manufacturer-recommended step, and where necessary, viability assessment was performed by staining cells with Zombie UV (BioLegend #423107; diluted 1:1000 in FACS buffer) or ViaDye Red (Cytek #R7-60008; diluted 1:500 in FACS buffer) to mark and exclude dead cells.
Instrument	All flow cytometry samples were analysed using an Aurora (Cytek), FACSymphony A3 (BD Biosciences), or LSR II (BD Biosciences).<br>Cell sorting was perforemd using an Aria III or an Aria Fusion (BD Biosciences).
Software	Flow cytometry data was captured using FACSDiva software (BD Biosciences, v8.0+).<br>Flow cytometry data was analysed using FlowJo (BD Biosciences, v8.0+).

Cell population abundance	Abundance varies by experiment. Representative graphs showcasing this data (where relevant) are provided in supplementary data.
Gating strategy	Gatign strategies vary by expeirment. For all relevant experiments, representative gating strategies are shown (e.g. Fig. S1E, S4A, S9A, S10A.

☒ Tick this box to confirm that a figure exemplifying the gating strategy is provided in the Supplementary Information.
